# Nonfouling textiles with tunable antimicrobial activity based on a zwitterionic polyamine finish†

**DOI:** 10.1039/c8ra09975h

**Published:** 2019-03-28

**Authors:** Lisa Maria Timma, Laura Lewald, Franziska Gier, Lisa Homey, Christian Neyer, Anna Nickisch-Hartfiel, Jochen Stefan Gutmann, Markus Oberthür

**Affiliations:** German Textile Research Centre North-West (Deutsches Textilforschungszentrum Nord-West) gGmbH 47798 Krefeld Germany timma@dtnw.de; Faculty of Chemistry, University Duisburg-Essen 45141 Essen Germany jochen.gutmann@uni-due.de; Center for Nanointegration Duisburg-Essen (CENIDE) 47057 Duisburg Germany; Faculty of Chemistry, Hochschule Niederrhein, University of Applied Sciences 47798 Krefeld Germany

## Abstract

Antimicrobial finishes for textiles and other surfaces that act without the release of biocides to the environment (contact biocides) or by inhibiting microbial adhesion (antifouling action) are viewed as promising and environmentally friendly alternatives to current products. We have used polyvinylamine polymers that were functionalized with zwitterionic sulfobetaine side chains with different degrees of substitution (DS) for the finishing of poly(ethylene terephthalate) (PET) and cotton fabrics in a water-based pad-dry-cure process. After washing with different surfactants, a stable finish with total polymer add-ons of 0.2–0.5 wt% was achieved. The finished textiles efficiently inhibited the adhesion of proteins and bacteria to the surface even with a small DS as low as 20%. Textiles finished with polymers with a low DS also showed significant antibacterial activity, most notably against *Staphylococcus aureus*. Accordingly, textile finishes with either pure antiadhesive (DS > 50%) or combined antiadhesive and antibacterial properties (DS = 20–50%) are accessible using this approach.

## Abstract 2

Depending on the degree of substitution DS, the antiadhesive (high DS) or antimicrobial (lower DS) character of the coating will be dominant.

## Graphical abstract

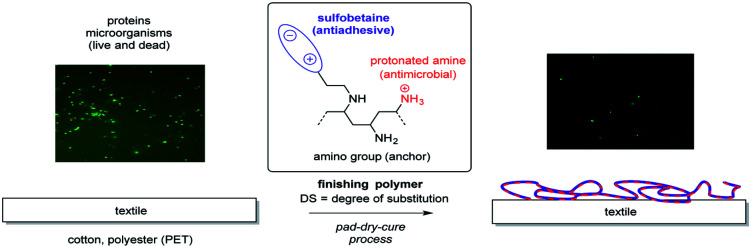

## Introduction

The design and implementation of surfaces with antibacterial properties is an important field of study that has moved beyond fundamental research on model surfaces to practical applications. In addition to medical and hygienic items (wound dressings,^1^ surgical sutures,^2^ stents and implants,^3^ equipment and interior objects in hospitals^4^), antimicrobial coatings are common features of widely used consumer goods and objects of daily life, *e.g.* digital hardware and equipment (touchscreens, keyboards, *etc.*), household appliances (refrigerators, washing machines, *etc.*) and food packaging.^5,6^ Other important fields of application are paints and finishes for surfaces that are prone to biofouling (sanitary installations, wallpaper, building fronts, ship hulls, *etc.*).^7^ Finally, antimicrobial finishes can also impart beneficial properties to a variety of textiles.^1,8^ For apparel and home textiles, the main purpose of the finish is to either improve the wear comfort (apparel) and freshness (apparel, home textiles) or to prevent the spreading of harmful pathogens, *e.g.* in hospitals and nursing homes (workwear, bedding, towels, pillows, mattresses, *etc.*). Antimicrobial finishes also improve the operating times and performances of technical textiles (filters, belts, canvasses, linings, *etc.*), because microbial growth can lead to optical, haptic and mechanical defects.^9^ Currently, various antimicrobial finishing agents for textiles are available from different suppliers. The most common finishes are based on silver compounds (silver salts, silver zeolites, nanosilver, *etc.*) and rely on the detrimental effect of silver ions on the growth of common bacteria.^12–14^ Because silver is less effective against fungi and higher organisms, the use of copper salts can be advantageous, particularly for antifouling applications and for material preservation.^15,16^ Other common antimicrobial agents for textiles are quaternary ammonium compounds, zinc pyrithione, polyhexamethylene biguanide (PHMB), and isothiozolinones.

Despite their effectiveness, textiles and other consumer products with antimicrobial activity are viewed critically by environmental organizations and regulatory and legislative bodies worldwide. It is feared that (a) the biocides (or degradation products) released can be harmful to humans and/or the environment and (b) the excessive use of biocides fosters the prevalence of resistant microorganisms. This assessment has manifested itself not only in stricter regulations regarding the use of biocides, but is also reflected in the buying patterns of consumers. Accordingly, there is an ongoing economic interest in the development of new antimicrobial technologies that do not rely on the release of traditional biocides.^10^ A promising approach in this context comprises the use of contact biocides, as they do not leach into the environment and act by a rather unspecific mechanism, *e.g.* cell wall destabilization by electrostatic interactions. In the textile sector, studies with polyamines and hydrophobic or quaternized derivatives, *e.g.* based on chitosan, polyvinylamine, or polyethyleneimine, have shown that good antibacterial activities can be achieved on fabrics with negligible leaching of the polymer or some other component of the finish to the environment.^11^ Another interesting approach, which has been studied in numerous model systems, is the use of antiadhesive surface coatings that do not necessarily kill microorganisms but prevent the attachment of proteins, bacteria, fungi and higher organisms. In this context, polyethylene glycol (PEG) based coatings on various substrates proved to be highly effective. The PEG groups on the surface are strongly hydrated, which leads to a well-ordered, hydrophilic surface layer that limits interactions with the surface itself. Other well-known strongly hydrated surface coatings are based on zwitterions (betaines), which were also able to reduce the adhesion of proteins, bacteria and fungi efficiently.^17–19^ PEG has also been used successfully to create textiles with antifouling properties. Because PEG is degraded oxidatively in the presence of light,^20^ however, zwitterionic surfaces are more promising targets for applications in the textile sector. Indeed, there are already reports about the modification of cotton with sulfopropyl betaines,^21,22^ which provide evidence that this technology is applicable to complex three-dimensional structures like fabrics. Nevertheless, different problematic issues remain with regard to a future industrial application, *e.g.* the stability of the siloxane-based sulfobetaines employed and the wash fastness of the resulting finishes,^21^ the use of organic solvents for the finishing step,^21,22^ and the limitation to cotton as a substrate. Accordingly, improved zwitterionic surface coatings for cotton and synthetic fibers, most importantly polyethylene terephthalate (PET), that are water-based, easily applied and durable, are still an important research goal. Usually, a one-pot process is used for the preparation of such coatings. For this purpose, either an auxiliary polymer is attached to the surface, at which subsequently antifouling compounds are grafted on or the antifouling compounds are grafted directly onto the surface. If, in addition, an antimicrobial effect is to be achieved, metal compounds are usually incorporated into the layer, which come into contact with the microorganisms and which kill microorganisms after leaching from the coating.^25^

Finally, it is hard to predict if antiadhesive zwitterionic polymers and surface coatings also act as antimicrobials, *i.e.* if the growth of microorganisms is actually impeded. There are some studies that found such activities,^21,22^ whereas others reported only antiadhesive properties.^23,24^ In general, a combined effect would be beneficial for many applications, because dead microbes tend to accumulate on contact-active surfaces, which leads to deactivation. For other applications and commercial products, a simple antiadhesive mode of action might be better suited, *e.g.* if a biocide-free product is desired. With this in mind, an easily tunable system that can provide both type of activities based on simple structural variations would be highly advantageous. So far, coatings based on surface groups that can switch between antimicrobial (cationic) and antiadhesive (zwitterionic) states *via* reversible saponification/esterification processes have been designed, but to the best of our knowledge, a zwitterionic surface that combines both activities based on a coating process with a single polymer has not been obtained yet.

**Fig. 1:**
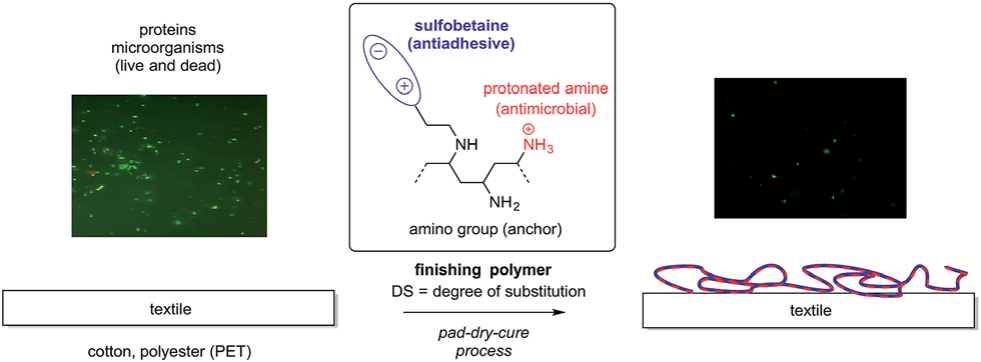
Finishing of textiles with zwitterionic polymers. Depending on the degree of substitution DS, the antiadhesive (high DS) or antimicrobial (lower DS) character of the coating will be dominant. The amino groups can either directly react with the fiber surface (PET) or serve as an anchor for a cross-linker, *e.g.* a triazine.

Here we present a flexible, durable finishing method for cotton and PET that offers anti-adhesive properties against proteins and microorganisms (Fig. 1). The special feature of our process is the possibility to precisely control the antiadhesive and antibacterial properties of the coating *via* the number of zwitterionic groups attached to the finishing polymer polyvinylamine as a result, the mode of action can be adjusted to the desired application. The finishing polymers are easily accessible by Michael addition by synthesis, and can then be applied in a water-based pad-dry-cure process which is commonly used for industrial textile finishing.

### Synthesis of zwitterionic polyamines with varying degree of substitution (DS)

The general design of the finishing polymers is based on two structural motifs. The antiadhesive activity is mediated by betaines (zwitterions) that are grafted to the primary amino groups of a polyamine (Fig. 1). The antimicrobial activity is based on the presence of positive charges (the protonated amino groups) in the polymer backbone itself. Because these amino groups also serve as anchors for the betaines, the antimicrobial activity should depend on the degree of substitution (DS) of the polymer, as the positive charges are “shielded” by the zwitterionic head groups, thereby limiting their interaction with the cell wall of bacteria and fungi. Accordingly, finishes based on polymers with a high DS should be purely antiadhesive, whereas finishes with a combined antiadhesive and antimicrobial activity can be accessible if polymers with lower DS are employed. It should be noted, however, that for different substrates, the optimal DS might depend on the fiber type (cotton, PET, *etc.*) and the specific textile structure (woven, knitted, *etc.*) employed. Finally, the remaining primary amino groups of the polymers serve as reactive anchors for the covalent linkage between the polymer and textile surface, either directly or *via* an additional cross-linker.

For our studies, we employed polyvinylamine (PVAm), which has been successfully used for the modification of different fiber types in various textile applications before. PVAm is commercially available in bulk quantities from different commercial suppliers under the trade name Lupamin®. Because it is already used in industrial processes, *e.g.* in the paper industry and for large-scale wastewater treatment, Lupamin® is available at comparatively low prices, which makes it an ideal starting material for the development of cost-competitive textile finishes. Lupamin® 9095 (*M*_w_ = 340 kDa) was found to be sufficiently pure to be used in the modification reactions directly.^26^

To obtain polyamines with varying degree of substitution (DS), we employed a Michael reaction of sulfobetaine acrylates SB1 and SB2 with Lupamin® 9095 in water (pH 11, 90 °C, 24 h). The syntheses of both betaines followed slightly modified published procedures^27^ and provided SB1 and SB2 in good yields. In the course of our studies, we could show that water proved to be the solvent of choice for the Michael reaction, as the coupling reactions with polyvinylamine proceeded much more efficient compared to reactions in organic solvents like methanol. In addition, it became apparent that acrylic Michael acceptors are preferable, as coupling reactions with the sulfobetaine methacrylate SBMA were sluggish and provided products that were hard to purify.^28^

**Scheme 1:**
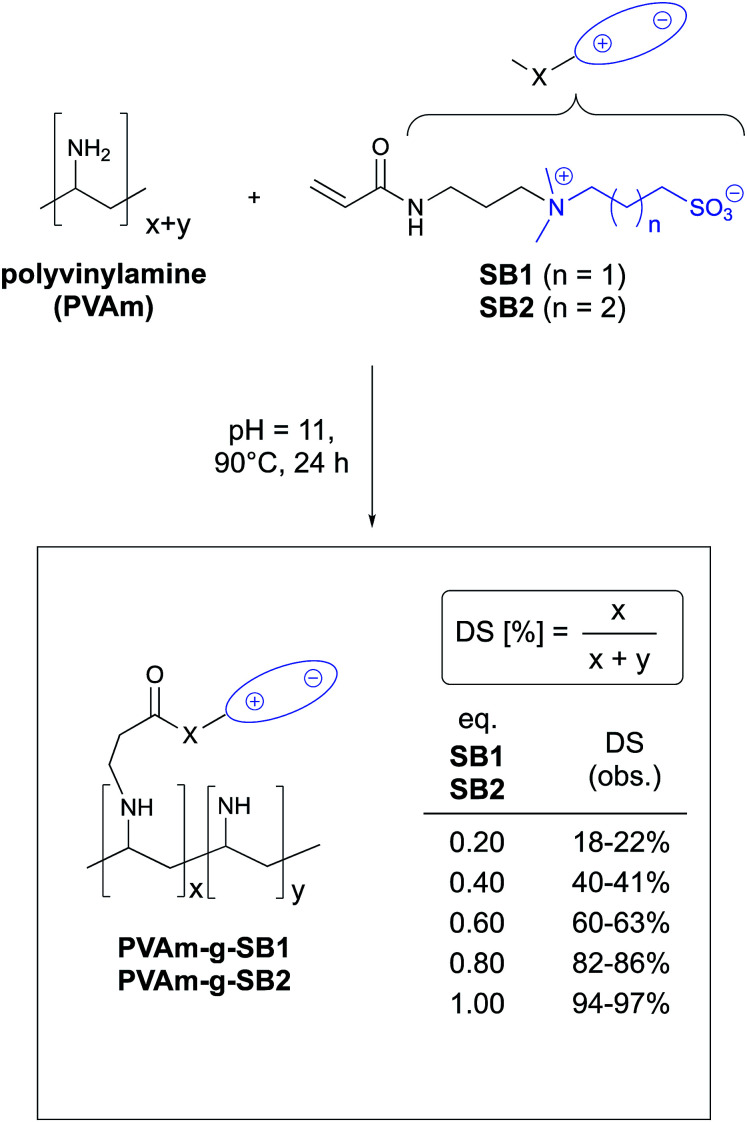
Reaction of PVAm with sulfobetaine acrylates SB1 and SB2. The range of DS (degree of substitution) of the resulting graft polymers that is shown on the right represents the results of multiple experiments.

For each conversion, the DS of the resulting product was determined by comparison of the signal intensity of suitable peaks in the ^1^H NMR spectrum (see ESi; Fig. S1–S6†) similar to previous reports.^29^ For all ratios tested, the DS corresponds well with the amount of SB1 and SB2 that was used (see inset in Scheme 1).^30^ Hence, in reactions of PVAm with acrylic sulfobetaines, the DS is easily controllable by the amount of acrylate monomer employed and can be increased to >90%. In addition, ^1^H NMR spectra of the crude reaction mixtures showed no remaining signals of the acrylate monomers. Because the coupling reactions proceed quantitatively, the resulting polymer solutions can be used directly (after dilution to 1 wt%) for the ensuing finishing process, which is an important advantage for large-scale production. For simplicity, the PVAm derivatives PVAm-g-SB1 and PVAm-g-SB2 used in the experiments described below are labeled according to the maximum DS that is theoretically possible (*e.g.*PVAm-g-SB1_20 when 0.2 eq. of SB1 was reacted with PVAm), as the actual DS of individually prepared batches during the course of the experiments differed slightly.^30^

### Finishing of different textiles with zwitterionic polyamines

**Scheme 2:**
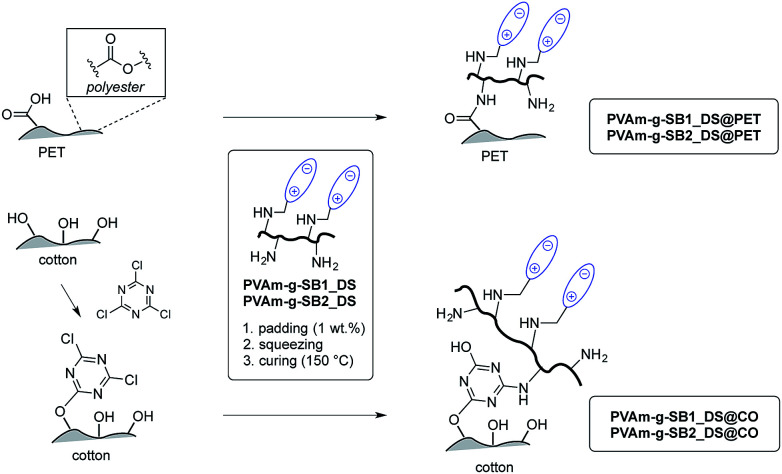
Finishing of polyester (PET) and cotton (CO) with sulfobetaine-modified PVAm. Whereas PVAm can be covalently linked to PET directly by heating, a triazine-based anchor was used for cotton fabrics. The DS of the polymers varied from 20–80%.

The sulfobetaine-containing PVAm derivatives synthesized in this study still contain unmodified primary amino groups that can serve as anchors for a permanent immobilization on different fiber types. It is known that PVAm can be linked covalently to PET fabrics without the addition of a cross-linker or a binder by curing at 140–150 °C through trans-amidation.^31,32^ At these temperatures, the primary amino groups of the polyamine can react either with the carboxylic acids present as end groups and with accessible ester groups at the surface of the PET fiber. In either case, an amide bond is formed, which is stable under a variety of conditions relevant for textiles (Scheme 2).^33,34^ Accordingly, PET fabrics were finished with PVAm-g-SB1 and PVAm-g-SB2 with varying DS by immersion into a 1 wt% polymer solution followed by squeezing (wet pick-up *ca.* 90–110% by weight). Afterwards, the fabrics were fixed at 150 °C in a lab-dryer using an adjustable pin frame.

For different cotton fabrics tested, finishing with PVAm-g-SB1 and PVAm-g-SB2 is possible, but the thermal fixation process was found to be less efficient, presumably because only a small number of carboxylic acid groups is present on the fiber surface (as a result of natural oxidation processes). Therefore, cotton fabrics were pretreated with cyanuric trichloride (1 g L^−1^ in acetone, room temperature), which functions as a cross-linking agent between the hydroxyl groups of the cellulose and the amino groups of the polymer. After air-drying, the same finishing sequence as described for PET fabrics was applied.

### Determination of the polymer add-on and washing fastness tests

**Fig. 2:**
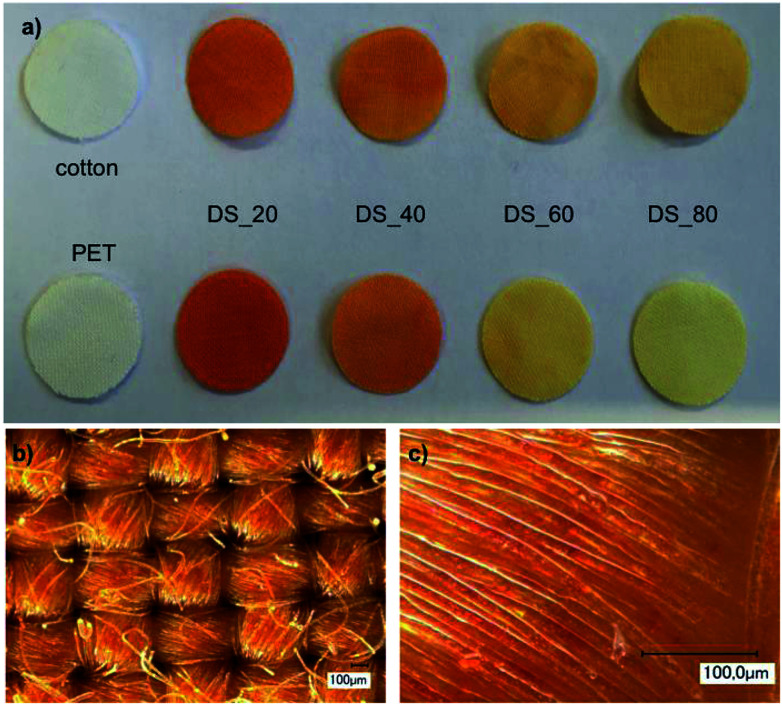
Characterization of the polymer add-on: (a) photographs of textiles (cotton and PET) finished with PVAm-g-SB2 with varying DS that were stained by TNBS; (b) and (c) light microscopic pictures of PET finished with PVAm-g-SB2_20 after TNBS staining.

The successful modification of the fabrics was confirmed qualitatively by staining with TNBS (2,4,6-trinitrobenzenesulfonic acid), which is selective for primary amino groups (Fig. 2). As shown in Fig. 2(a), the intensity of the TNBS stain of PET and cotton fabrics finished with PVAm-g-SB2 decreases with increasing DS, as the number of primary amino groups also decreases. The light microscopic pictures in Fig. 2(b) and (c) provide evidence that the polymer coating covers the fiber surface rather evenly with the exception of the small areas where the warp and weft threads overlap. This was corroborated by SEM-EDX measurements, which showed an even distribution of sulfur on the fiber surface (data not shown). The most reliable method to assess the total amount of polymer attached to the fabrics quantitatively proved to be the determination of the sulfur content of hydrolyzed samples using ICP-OES (inductively coupled plasma optical emission spectroscopy). The gravimetric determination of the add-on, on the other hand, was difficult because of the strong hydration of the fiber surface (see ESI; Fig. S7†). We also tried to record IR spectra to detect the polymer layer. However, neither with a KBr-compact nor with the ATR technique could a spectrum be recorded in which the resulting amide bands were visible. The polymer add-on is therefore too low to get a meaningful IR-spectra.

**Fig. 3:**
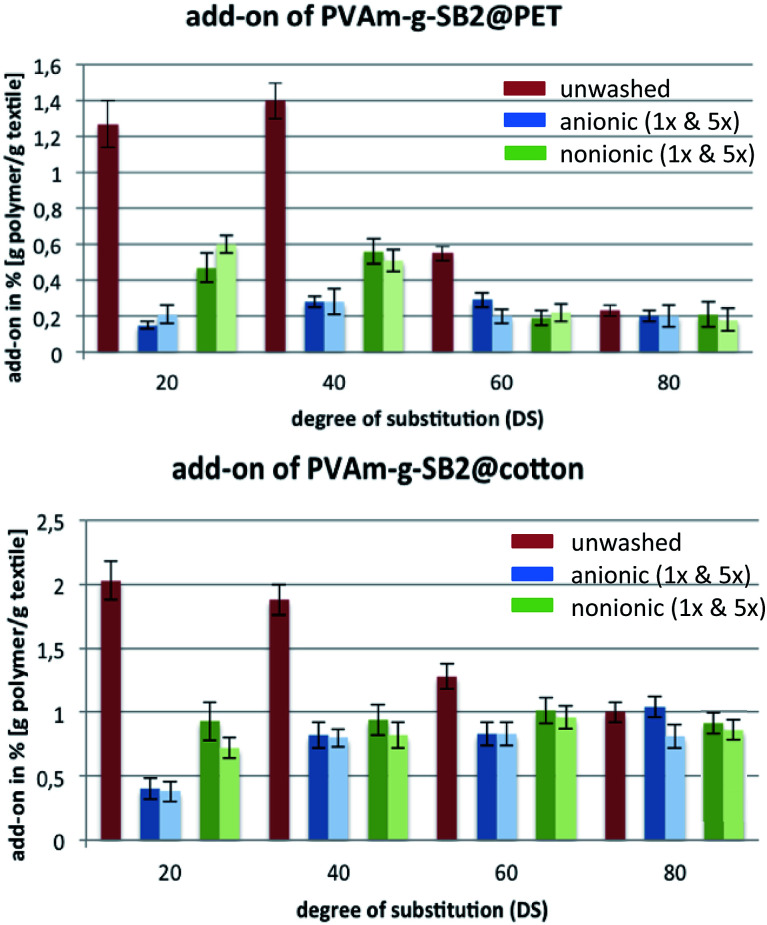
Quantitative determination of the add-on of PVAm-g-SB2 on PET and cotton by ICP-OES. After the finishing step, textiles were thoroughly rinsed with water (no detergent, red columns) and subsequently washed either with anionic (blue columns) or nonionic (green columns) detergents. Number of wash cycles: 1× (dark), 5× (pale).

It is difficult to determine the layer thickness. It could only determined a theoretical layer thickness with the add-on (determined by ICP-OES) and the surface (smooth) but it is not meaningful, because the exact surface of the fibers in the yarn or tissue composite is unknown. Directly after the fixation step, the add-on was determined by ICP for samples that were thoroughly rinsed with water, as shown in Fig. 3 for PVAm-g-SB2 (red bars). For cotton samples, a higher add-on was observed in comparison to PET, which can be attributed to the use of the cross-linker. Washing fastness tests of the finished textiles^35^ were then conducted with different sulfur-free surfactants (anionic: sodium stearate, blue bars; and nonionic: alkylated poly(ethyleneglycol), green bars) to make sure that small amounts of adhering detergent did not distort the ICP results. The analysis of the obtained data showed that for both PET and cotton samples, the sulfur content decreased quite substantially after one wash cycle with either surfactant (dark blue and green bars, respectively) when polymers with a DS < 80% were used for the finishing step.

For polymers with low to medium–high DS values, a large portion of the initially adsorbed polymer is only loosely bound to the fiber surface and can be removed by surfactants. The use of anionic surfactant is more effective for lower DS and removes more polymer from the surface than nonionic surfactant, which is probably due to the interaction of the ionic groups. For polymers with a large number of zwitterionic groups, the add-on is initially much smaller (*e.g.* on PET: 0.2% for DS = 80% *vs.* 2% for DS = 20%), but decreases only slightly after the treatment with the surfactants, which means that unspecific adsorption is not a factor in these cases. Independent of the DS, however, additional wash cycles (up to five) did not result in a further decrease of the sulfur content (light blue and green bars, respectively). This shows that the surface coating which remains after the first wash step is tightly bound to the fiber and can withstand multiple wash cycles. Similar results were obtained for PVAm-g-SB1-based finishes (see ESI; Fig. S8–S11†).

### Surface morphology of textiles finished with PVAm-g-SB2

**Fig. 4:**
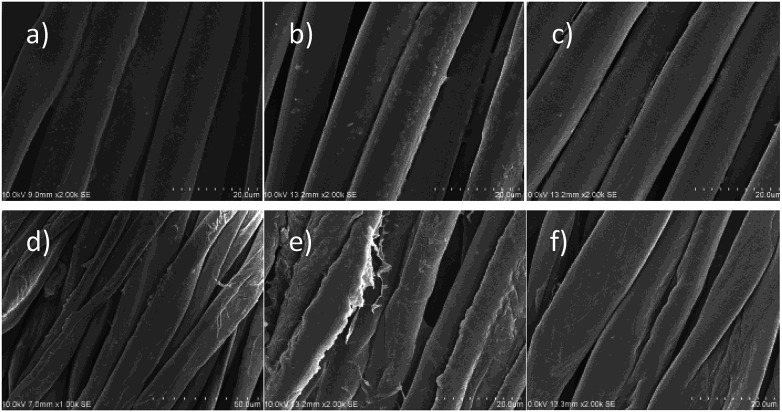
SEM images of fabrics before and after finishing with PVAm-g-SB2_20. (a) PET untreated; (b) PET directly after the finishing step; (c) PET after finishing and washing with ECE detergent; (d) cotton untreated; (e) cotton directly after the finishing step; (f) cotton after finishing and washing with ECE detergent.

Fig. 4 shows SEM images of PET and cotton fabrics that were finished with PVAm-g-SB2_20. For PET, there are only small deposits observable directly after the finishing (Fig. 4(b)) compared to untreated PET (Fig. 4(a)). After the finished fabric was washed with ECE detergent (Fig. 4(c)), the fiber surface appears very similar to the unfinished fabric, which is in agreement with the low add-ons determined (see Fig. 3). For cotton fabric, a more extensive and rather even polymer coating is visible directly after the finishing step (Fig. 4(e)). After the treatment with detergent (Fig. 4(f)), this coating is no longer present, and the fiber surface appears smoother than untreated cotton (Fig. 4(d)).

### Protein adhesion studies

The finished textiles were then used in protein adhesion studies. The attachment of proteins on surfaces is not only a simple experimental model for microbial adhesion. Moreover, it is also the first step in the formation of bacterial biofilms, which are hard to eliminate and support the fouling of higher organisms to surfaces. For the adhesion experiments, a lipase (type VII from *Candida rugosa*) that is able to cleave fluorescein diacetate into fluorescein was used as a model protein. The fluorescence emission of the dye liberated can then be used to quantify the amount of protein that is attached to the surface of the textile. To study the effect of different surfactants on the protein adhesion in general, PET and cotton reference textiles were washed with the anionic and nonionic surfactants used for the wash stability tests as well as the so-called ECE detergent, which is a mixture of neutral and anionic surfactants, and were then tested in the protein adhesion assay. Whereas washing the samples with either alkylated PEG or ECE detergent did not influence the lipase adhesion, a substantial increase was observed for the anionic detergent sodium stearate (see ESI; Fig. S12†), especially for cotton. As a consequence, ECE detergent was chosen for the subsequent test with finished fabrics because of its similarity to common household detergents.

**Fig. 5:**
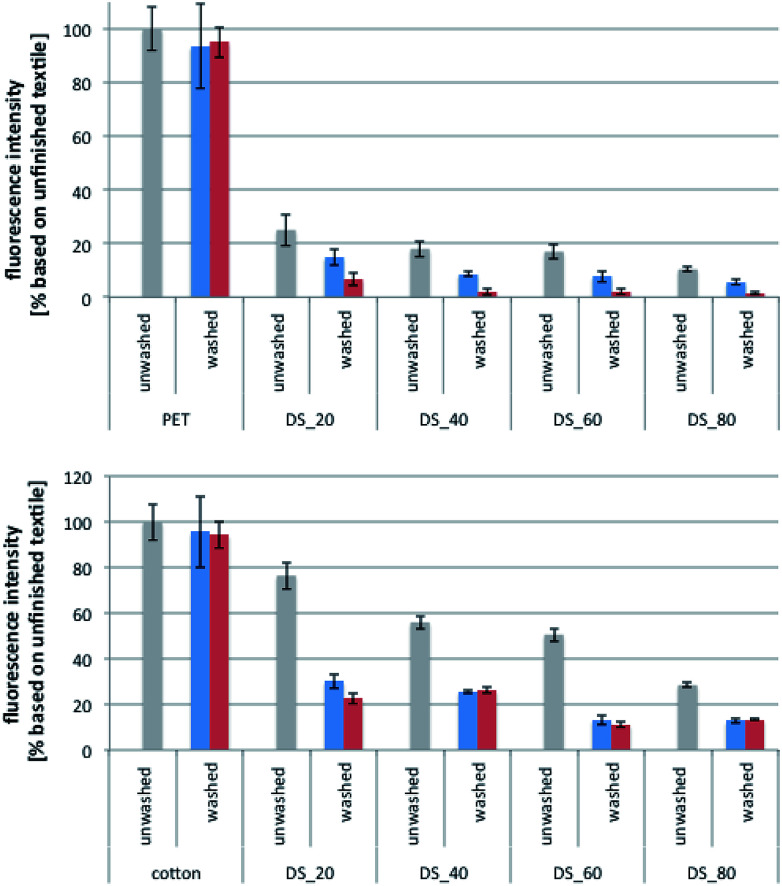
Results of the protein adhesion tests for different fabrics (PET and cotton). The fabrics were finished with sulfobetaine-modified PVAm-g-SB2 with different degrees of substitution (DS) and then used either directly after rinsing with water (unwashed) or after 1 wash cycle (blue columns) and 5 wash cycles (red columns) according to DIN EN ISO 105-C06 using ECE detergent.

From the results of the protein adhesion assay (Fig. 5), three main conclusions can be drawn. Firstly, PET and cotton samples that were finished with sulfobetaine-containing polyamines showed a significant reduction of protein adhesion. Whereas this general trend was anticipated, additional experiments showed that the antiadhesive properties of the finished fabrics actually improved after washing with detergent. Specifically, it was found that protein adhesion was lower for samples that were washed with ECE detergent (5 cycles, green columns) than for samples that were only briefly rinsed with water (blue columns), *e.g.* when PVAm-g-SB2 was used as the finishing agent. The amount of adsorbed lipase dropped primarily after the first wash cycle (see ESI; Fig. S13†), which, according to the results of the wash fastness tests (Fig. 2), is also responsible for the removal of the largest proportion of loosely bound polymer. Accordingly, the results of the protein adhesion assay provide evidence that the tightly bound surface layer, which becomes better accessible after the first wash cycle with the surfactant, provides superior antiadhesive properties compared to the initial coating.

Secondly, whereas the antiadhesive properties of the finished textiles get better with an increasing degree of substitution, significant effects can already be detected for small DS values. For polyester, *e.g.*, even polymers with a sulfobetaine content of DS = 20% were able to reduce the level of protein adhesion to below 10% (after 5 wash cycles). Polymers with a higher DS further reduced the amount of lipase adhesion to about 5%, but more or less independent of the exact DS value. For cotton samples, protein adsorption decreased less for DS = 20% (*ca.* 25% protein adhesion compared to the reference sample), and the threshold for a further reduction to about 15% was shifted to higher substitution ratios of 50% and more. Samples finished with PVAm-g-SB1 showed a similar trend in the protein adhesion assay but were slightly less efficient (see ESI; Fig. S14†).

**Fig. 6:**
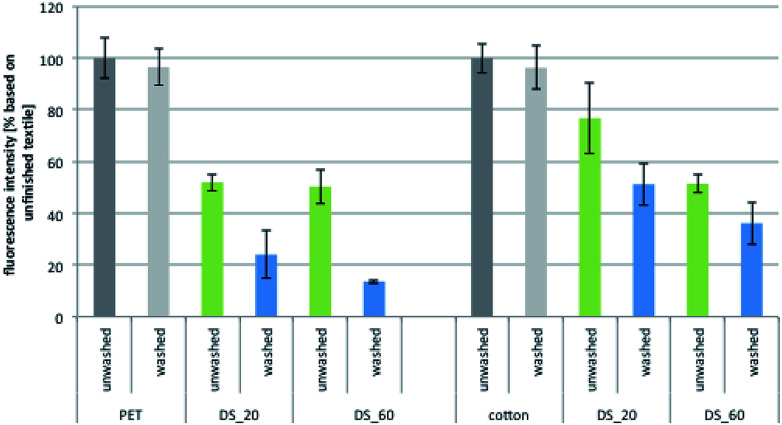
Results of the protein adhesion tests for PVAm-g-PEGMA on PET and cotton. The fabrics were finished with different degrees of substitution (DS) and then used either directly after rinsing with water (unwashed) or after 5 wash cycles according to DIN EN ISO 105-C06 using ECE detergent (washed).

Thirdly, the reduction of protein adhesion observed for the sulfobetaine-PVAm finished textiles compares well with PEG-based coatings and can even lead to superior results. For comparison, we synthesized PVAm-g-PEGMA with 9 ethyleneglycol repeating units by the reaction of Lupamin® 9095 with the corresponding polyethyleneglycol methacrylate (PEGMA), which was then used for finishing PET and cotton fabrics (see ESI†). Because of the high gelation tendency of these PEG derivatives, the purification and exact analysis of the products by NMR spectroscopy was difficult. Therefore, the DS of the PVAm-g-PEGMA derivatives used for coating experiments (20% and 60%) were calculated based on the assumption that the methacrylate monomers added to the reaction mixture reacted completely with the polyamine, which seemed acceptable because no remaining monomer could be detected in the crude reaction mixtures. For PET, the reduction of protein adhesion by the two PEG-based coatings was less efficient compared to the coatings with PVAm-g-SB2 of similar DS. For cotton, the difference was less pronounced, but still observable for the washed samples. In addition, the effect of the washing cycles was not consistent, and did not reduce protein adhesion as pronounced as for the sulfobetaine polymers (Fig. 6).

Thus, it can be summarized that the sulfobetaine containing polyamines are (1) easier to synthesize and purify, and (2) in general perform better than the corresponding PEG derivatives, especially for washed samples. Accordingly, all further studies were conducted with PVAm-g-SB2.

### Inhibition of bacterial adhesion

**Fig. 7:**
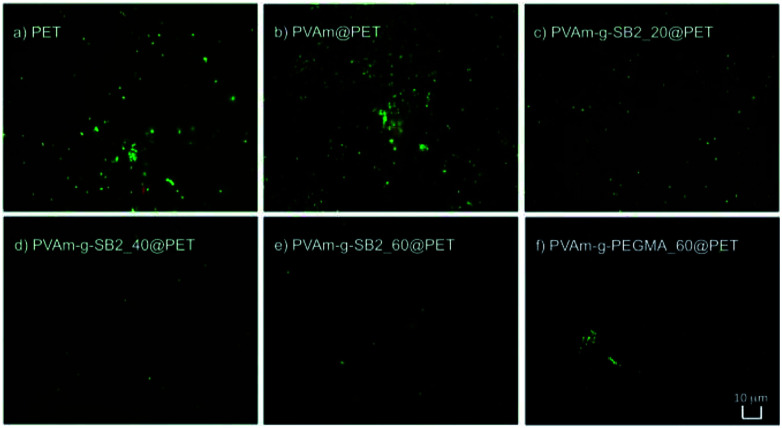
Fluorescence micrographs of different polyester fabrics after incubation with *E. coli* cell suspensions and staining (green: live, red: dead). (a) Untreated; (b) finished with PVAm; (c–e) finished with sulfobetaine-modified PVAm with DS 20, 40, and 60%; (f) finished with PEG-modified polyvinylamine with DS = 60%.

Experiments with *Escherichia coli* as a model organism provided evidence that PVAm-g-SB2 finished textiles were also able to reduce microbial adhesion. After immersion into a bacterial cell suspension, cells adhering to the textile surface were visualized using a live–dead stain with the dyes SYBR green 1 (green, live cells) and propidium iodide (red, dead cells), respectively. The fluorescence microscopic images of polyester fabrics (either unfinished or finished with PVAm, PVAm-g-SB2 polymers with different DS, and PVAm-g-PEGMA as a reference) that were treated according to this procedure are shown in Fig. 7. The comparison of the unmodified PET fabric (Fig. 7(a)) and the fabric finished with PVAm (Fig. 7(b)) shows that unmodified PVAm does not reduce the amount of living bacteria adhering to the fabric. Although PVAm is considered to act as a contact biocide, there are also not many dead cells observable. This is due to the short contact times between the fabric and the bacterial suspension (30 minutes) in comparison to standard antibacterial assays (24 hours, see below). The images of the sulfobetaine-containing fabrics (Fig. 7(c–e)) clearly show a distinct reduction of bacterial adhesion with increasing DS compared to the untreated PET fabric. Very similar to the results of the protein adhesion assay, a drastic reduction of adhesion is already achieved with polymers with a small DS. The comparison with Fig. 7(f) (PEGMA) illustrates that bacterial adhesion for the sulfobetaine surfaces is similar to coatings based on PEG, which are the “gold” standard of antiadhesive coatings. Finally, very similar results were obtained for cotton fabric (see ESI; Fig. S15†), which shows that the antiadhesive polyamines used in this study are able to act as antifouling finishes for a variety of textile substrates.

### Antimicrobial activities

Tests according to JIS L 1902:2008 showed that only textiles finished with PVAm-g-SB2 with small to medium DS have antimicrobial activity against Gram-positive (*S. aureus*) and Gram-negative bacteria (*E. coli*).

**Fig. 8:**
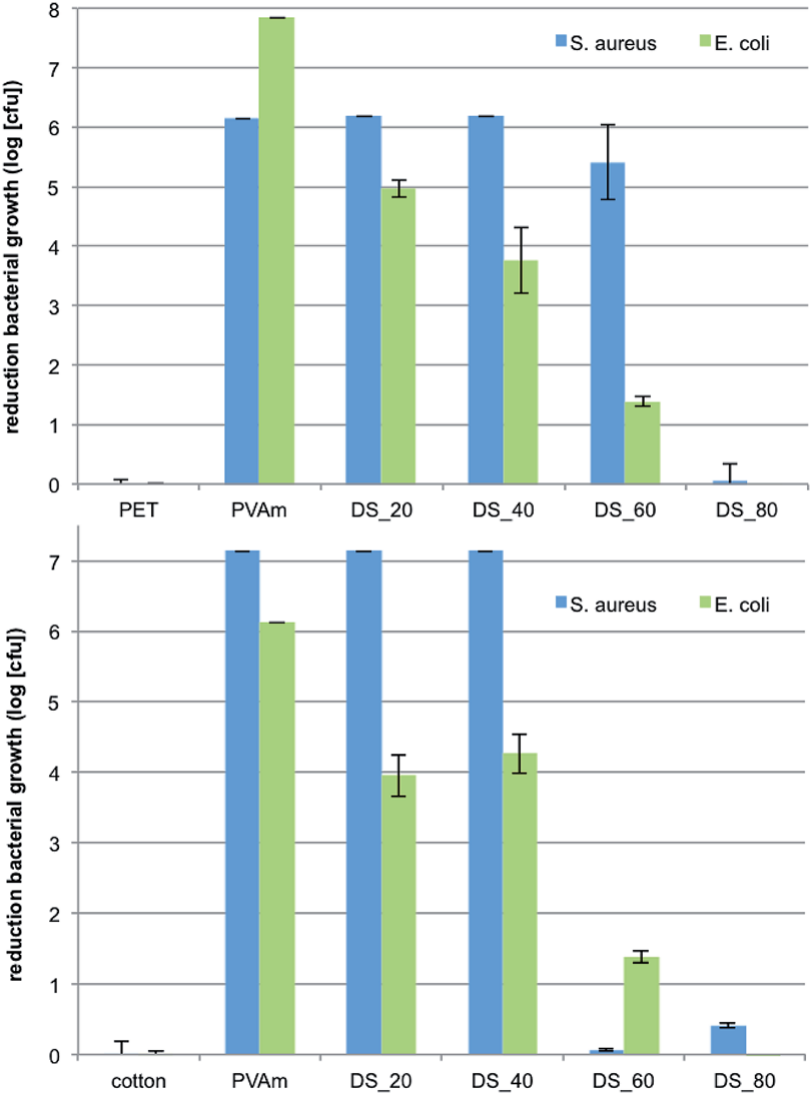
Antibacterial activity against the Gram-positive strain *S. aureus* and the Gram-negative strain *E. coli* of different PET (top) and cotton (below) fabrics modified with PVAmg-SB2 with different DS.

Polyester or cotton finished with polymers with a low degree of substitution (DS = 20%, *i.e.* with a large number of primary amines) showed an activity against *Staphylococcus aureus* that is similar to polyvinylamine itself (log 7 reduction, Fig. 8). Interestingly, the fabrics finished with PVAm-g-SB2 with DS = 20% and 40% were also still of high activity, whereas finishes based on polymers with a DS of 80% (*i.e.* with a small number of primary amines) lost almost all activity. Polymers with DS = 60% are intermediary, and their specific activity seems to be dependent on the type of fiber. For the Gram-negative strain *E. coli*, substitution of the amino groups has a more distinct adverse effect on antibacterial activity. In this case, polymers with DS = 20% were already less active, whereas polymers with a DS of 60% and higher showed only negligible activity.^36^ The results match very well with the data reported in previous work. It has been shown that Gram-positive strains are often easier to kill than Gram-negative strains because of their cell wall structure.^37,38^ The effect of the polymer DS on the other hand, which correlates with the grafting density of the zwitterionic groups on the textile fiber surface, has not been studied systematically previous to our studies to the best of our knowledge.

**Fig. 9:**
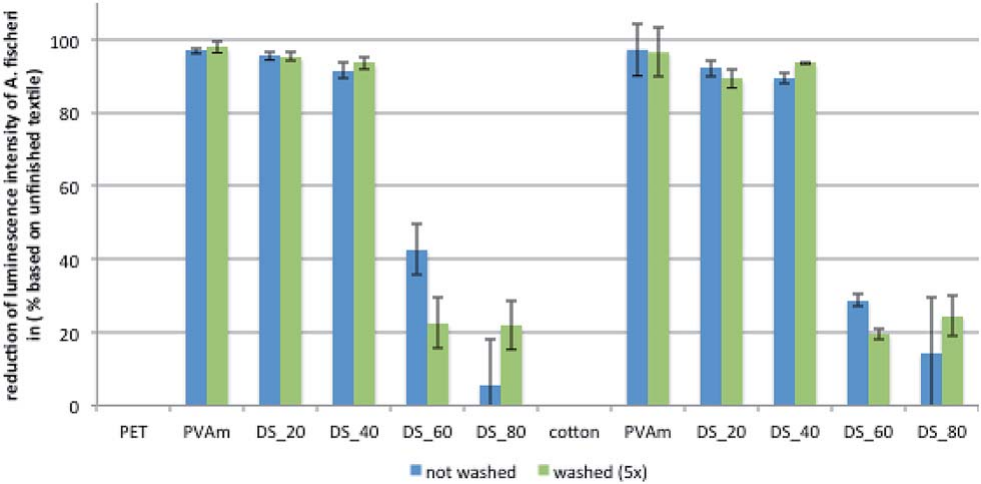
Antibacterial activity before and after 5 wash cycles against the Gram-negative strain *A. fischeri* of different PET and cotton fabrics modified with PVAmg-SB2 with different DS.

Finally, the washing fastness of the antimicrobial effect for the PVAm-sulfobetaine-based finishes was also determined, using a newly designed fluorescence assay with two Gram-negative strains, *E. coli pGLO* and *Aliivibrio fischeri*. After incubation in the bacterial solution, the fluorescence of the green fluorescent protein expressed by *E. coli pGLO* or the inherent luminescence of *A. fischeri* could be detected directly on the textile. The validity of the assay was first confirmed by comparison of the results obtained for *E. coli* (Fig. 9) with the results of the tests according to JIS L 1902: 2008 (Fig. 8), which revealed similar trends. As before, finished textiles with polymers with a DS of up to 40% showed a significant antimicrobial efficacy, whereas polymers with a higher DS provided only a minor reduction of bacterial growth.

After 5 wash cycles (green bars, Fig. 9), the antimicrobial activity of most textile samples was very similar to the non-washed samples so in contrast to the results of the protein adsorption experiments, washing has only a minor effect. Similar results were obtained for *E. coli pGLO* (see ESI; Fig. S18†).

These experiments suggest that the use of a contact active surface can lead to similar effects compared to the use of metal compounds as biocides. The antimicrobial effect of coatings based on metal salts usually decreases over time, as they are lost over time due to leaching. The washing tests have shown that after an initial loss of loosely associated polymers, the remaining coating is stable to multiple washing steps, which is promising for a variety of applications. Simple contact active surfaces often suffer a loss of activity because of biofouling. As our coatings show stable antiadhesive as well as antimicrobial properties, biofouling should be a minor problem. Long-term studies with finished textiles are currently underway and will be reported in due time.

## Experimental

### Finishing of textile samples

#### Pre-treatment of cotton

The samples were immersed for 5 minutes in a cyanuric chloride solution (1 g L^−1^ in acetone) and then dried in a fume hood.

#### Finishing of polyester and cotton by a pad-dry-cure process

For this purpose, the samples were immersed in an aqueous solution of (modified) polyamine (1 wt-%) and conditioned for 1 minute. The conditioned pieces of fabric were squeezed off with a padder, so that the liquor pickup was about 100%. Subsequently, the tissue was fixed in a labdryer using a tenter (8 min at 150 °C). Thereafter, the samples were each rinsed for 5 min with tap water and 5 min with deionized water to remove excess polymer. The washed samples were then dried at room temperature.

### Determination of free amino groups by TNBS staining

The textile samples were individually placed in small Petri dishes in an aqueous 2,4,6-trinitrobenzenesulfonic acid solution (0.1 wt%) and incubated for 5 min at 80 °C. Subsequently, the samples were rinsed for 5 minutes with water and then air-dried. The staining of the samples was visually inspected and recorded by photography.

### Determination of wash fastness

Wash resistance was determined according to DIN EN ISO 105-C06: 1997 (150 mL wash solution, liquor ratio 1 : 30, ECE detergent 4 g L^−1^, 30 min at 40 °C, 10 steel balls). For the determination of the polymer add-on of the textiles only single surfactants containing no sulfur (an anionic and a non-ionic detergent) were used, as most conventional detergents contain sulfur compounds, which could distort the result of the ICP-OES measurements. However, the samples, which were used for lipase adhesion, were washed with a standard detergent similar to DIN EN ISO 105-C06.

### Determination of protein adsorption

Textile samples (*d* = 15 mm) were incubated in a lipase solution (type VII from *Candida rugosa*, 1 g L^−1^ in 100 mM phosphate buffer pH = 7.2) and placed on a shaker at 50 rpm for 30 min. The samples were then placed on the shaker in deionized water for 1 minute to remove only slightly adhered lipase. To determine the amount of tightly bound lipase, the samples were then dried and transferred into a 24-well plate. 500 μL of phosphate buffer were added to each well. The plate was then placed in a TECAN plate reader with an injection unit. 50 μL FDA (2 mg mL^−1^ in DMF) were added to each well automatically, before the fluorescence intensity (absorption 485 nm, emission 535 nm) was measured after 15 s of shaking.

### Determination of bacterial adhesion

For live–dead staining, an *E. coli* culture was grown in LB medium. For this purpose, a colony was picked from an agar plate and dissolved in LB medium. Cultivation took place overnight in a shaker at 37 °C and 200 rpm. Subsequently, the textile samples (*d* = 15 mm) were incubated with the bacterial solution for 30 min on a rotary shaker at 50 rpm. After incubation, the textiles were removed from the bacterial solution, rinsed off briefly and transferred to an Eppendorf tube containing a mixture of 300 μL propidium iodide and 300 μL SYBR-Green for at least 5 minutes. The samples were then placed on microscopic slides and covered with coverslips before examination under the fluorescence microscope.

### Determination of antimicrobial activity

The determination of antimicrobial activities according to JIS L 1902:2008 was carried out by the *in vitro*-Research Laboratory of the Clinic of Dermatology, University Hospital Jena, Germany.

### Determination of antimicrobial activity after washing

#### Aliivibrio fischeri

Cultivation took place in seawater liquid medium (or on seawater agar plates) according to DSMZ composition (medium 246). 10 mL of the nutrient medium were inoculated with *A. fischeri* and incubated for 48 h at 17 °C. The luminescence of 100 μL preculture was measured in a 96-well plate and diluted so strongly with nutrient medium that the luminescence was about 100 000. The textiles were placed in a 24-well plate and supplied with 100 μL of a 2% NaCl solution. Subsequently, 100 μL of the diluted preculture were added and the luminescence was measured over a period of 24 h.

## Conclusion

We have developed functional finishes based on zwitterionic polyamines that are able to reduce the adhesion of proteins and bacteria to textiles significantly. In addition, it is possible to control the antimicrobial activity of the finishes by varying the degree of substitution of the functional polymers. Accordingly, it should be possible to optimize finishes for specific fibers and applications that either are purely antiadhesive or have a combined antiadhesive and antimicrobial activity. The synthesis of the functional polymers is straightforward and is amenable to scale-up to an industrial level. The finishing process itself is simple, water-based, and does not need sophisticated machinery, which will facilitate the transfer of this technology to an industrial process. Additional studies regarding the effect of the finishes on different textile properties, *e.g.* tear strength, water vapor and air permeability, and their biocompatibility are currently under way.

## Conflicts of interest

There are no conflicts to declare.

## Supplementary Material

RA-009-C8RA09975H-s001
