# Supplementary material for: Nonfouling textiles with tunable antimicrobial activity based on a zwitterionic polyamine finish
Source: RSC Adv. 2019 Mar 28;9(17):9783–91. doi: 10.1039/c8ra09975h (PMC9062160; doi:10.1039/c8ra09975h)
Supplement: RA-009-C8RA09975H-s001 [file RA-009-C8RA09975H-s001.pdf]

Electronic Supplementary Material (ESI) for RSC Advances.
This journal is © The Royal Society of Chemistry 2019

Supporting Information

Nonfouling textiles with tunable antimicrobial activity based on a zwitterionic polyamine finish

Lisa M. Timma,[a,b,c] Laura Lewald,[d] Franziska Gier,[d] Lisa Homey,[d] Christian Neyer,[d]

Anna Nickisch-Hartfiel,[d] Jochen S. Gutmann,[a,b,c*] Markus Oberthür[a,e*]

[a] *German Textile Research Centre North-West (Deutsches Textilforschungszentrum Nord-West) gGmbH, 47798 Krefeld, Germany. E-mail: gutmann@dtnw.de*
[b] *Faculty of Chemistry, University Duisburg-Essen, 45141 Essen, Germany.*
[c] *Center for Nanointegration Duisburg-Essen (Cenide), 47057 Duisburg, Germany.*
[d] *Faculty of Chemistry, Hochschule Niederrhein, University of Applied Sciences, 47798 Krefeld, Germany.*
[e] *Current address: Department of Design, Hochschule für Angewandte Wissenschaften Hamburg, 20087 Hamburg, Germany. E-mail:markus.oberthuer@haw-hamburg.de*

Analytical data for 1-propanaminium-*N*,*N*-dimethyl-*N*-{3-[(1-oxo-2-propen-1-yl)amino]-propyl}-3-sulfonat, inner salt (SB1)

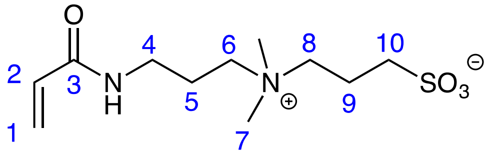

SB1

To a solution of *N*-[3-dimethylaminopropyl]acrylamide (**1**, 4.93 mL, 30 mmol) in acetonitrile (10 mL) was added a solution of propane-1,4-sultone (**3**, 3.66 g, 30 mmol) in acetonitrile (5 mL). The mixture was stirred at room temperature for 24 h with vigorous stirring. The solid was filtered by suction, washed with small aliquots of acetonitrile, and dried under vacuum. Sulfobetaine **SB2** (7.85 g, 95%) was obtained as a colorless, amorphous powder.

^{1}H NMR (300 MHz, D_2O): δ/ppm = 6.08-6.16 (m, 2H, 1-H_2), 5.75 (dd, 1H, J = 3 and 9 Hz, 2-H), 3.41-3.45 (m, 2H, 8-H), 3.33–3.40 (m, 4H, 4-H_2, 6-H_2), 3.10 (s, 6H, 2 x 7-H_3), 2.98 (t, 2H, J = 2 Hz, 10-H_2), 2.08-2.14 (m, 2H, 9-H_2), 2.00-2.06 (m, 2H, 5-H_2).

^{13}C NMR (75 MHz, D_2O): δ/ppm = 169.0 (C-3), 129.9 (C-2), 127.8 (C-1), 62.4, 62.2 (C-6, C-8), 50.9 (2 x C-7), 47.2 (C-10), 36.2 (C-4), 22.5 (C-5), 18.2 (C-9).

ESI-MS: [M]+ calcd. for $C_{11}H_{22}N_2O_4S$: 278.13, found: 279.1 [M]+

Synthesis and analytical data for 1-butanaminium-*N*,*N*-dimethyl-*N*-{3-[(1-oxo-2-propen-1-yl)amino]-propyl}-3-sulfonat, inner salt (SB2)

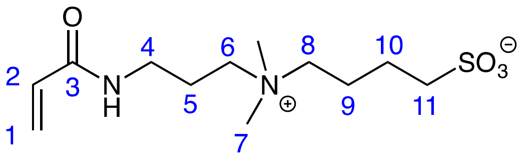

SB2

To a solution of *N*-[3-dimethylaminopropyl]acrylamide (**1**, 4.93 mL, 30 mmol) in acetonitrile (10 mL) was added a solution of butane-1,4-sultone (**3**, 4.69 g, 30 mmol) in acetonitrile (5 mL). The mixture was stirred at room temperature for 24 h with vigorous stirring. The solid was filtered by suction, washed with small aliquots of

acetonitrile, and dried under vacuum. Sulfobetaine **SB2** (6.58 g, 75%) was obtained as a colorless, amorphous powder.

^{1}H NMR (300 MHz, D_2O): δ/ppm = 6.10-6.17 (m, 2H, 1-H_2), 5.77 (dd, 1H, J = 3 and 9 Hz, 2-H), 3.25-3.40 (m, 6H, 4-H_2 , 6-H_2 , 8-H_2), 3.05 (s, 6H, 2 x 7-H_3), 2.95 (t, 2H, J = 2 Hz, 11-H_2), 1.97-2.03 (m, 2H, 15-H_2),1.82-1.88 (m, 2H, 9-H_2), 1.75-1.80 (m, 2H, 10-H_2).

^{13}C NMR (75 MHz, D_2O): δ/ppm = 169.0 (C-3), 129.9 (C-2), 127.8 (C-1), 63.5, 61.9 (C-6, C-8), 50.8 (2 x C-7), 50.1 (C-11), 36.2 (C-4), 22.3 (C-5), 21.0 (C-9, C-10).

ESI-MS: [M]+ calcd. for $C_{12}H_{24}N_2O_4S$: 292.15, found: 293.2 [M]+

Determination of the degree of substitution (DS) for reaction products with PVAm

The reaction products **PVAm-g-PEGMA** and **PVAm-g-SBMA** formed gels when dissolved in various deuterated solvents. Accordingly, the exact DS of these polymers could not be determined by NMR.

PVAm-g-SB1 and **PVAm-g-SB2**, on the other hand, were easily soluble in D_2O. For the determination of DS of **PVAm-g-SB1** (Figure S1), the ^{1}H NMR peaks of the methylene group of the PVAm backbone (A-H_2) and the methylene group (2'-H_2) formed by the Michael addition were used, as they were not overlapping with other signals. The DS is equivalent to the ratio of the two integrals multiplied by 100%.

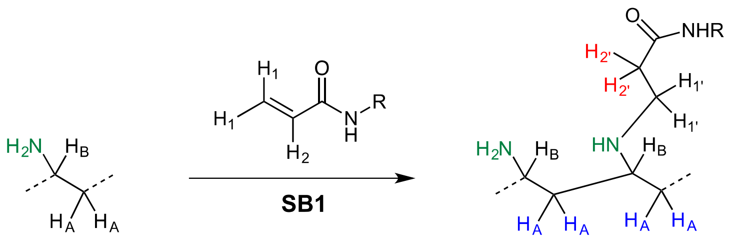

polyvinylamine (PVAm) **PVAm-g-SB1**

$$DS = \frac{\text{integral (2-H}_2\text{)}}{\text{integral (A-H}_2\text{)}} \times 100\%$$

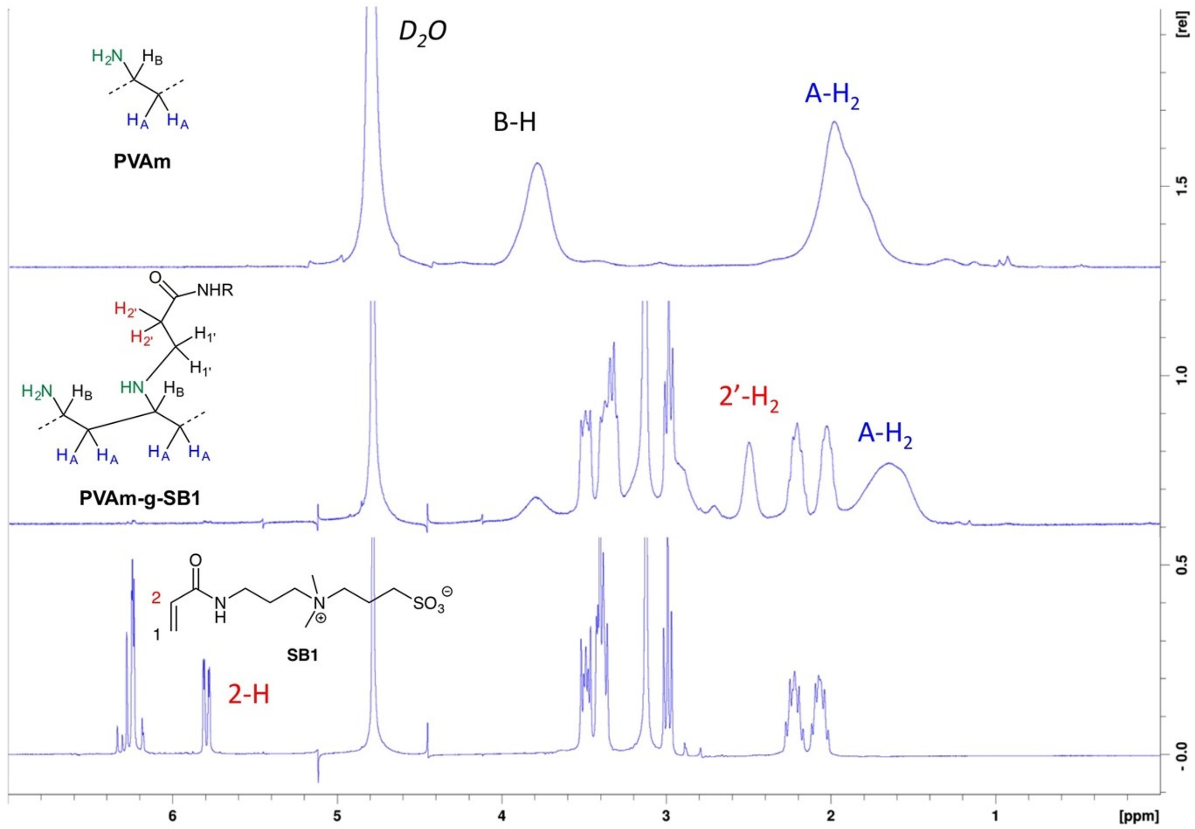

Figure S1. Determination of DS for **PVAm-g-SB1** and ^{1}H NMR spectra (in D_2O, 300 MHz) of PVAm, **PVAm-g-SB1** and **SB1**.

For the determination of DS of **PVAm-g-SB2** (Figure S2), the ^{1}H NMR peaks of the methylene group of the PVAm backbone (A-H_2) could not be used because other signals were overlapping. Instead, the signal of the methine groups (B-H) and the methylene group (2'-H_2) were used. The DS is equivalent to the ratio of the two integrals multiplied by 0.5 (1/2 H atom ratio for each repeating unit) and 100%.

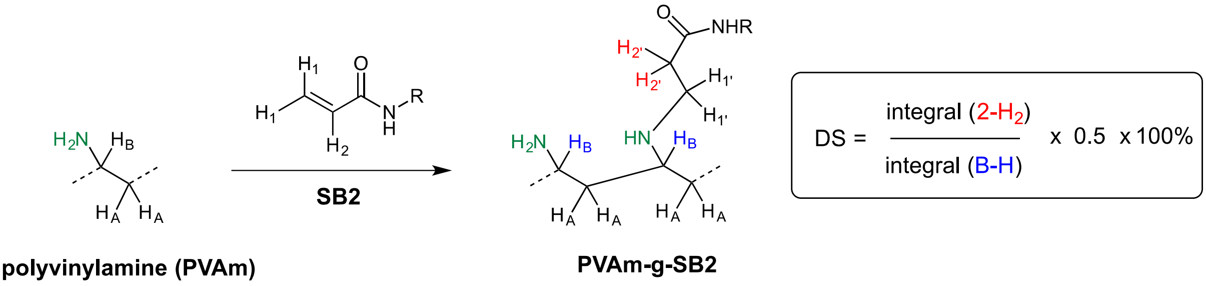

Figure S2. Determination of DS for **PVAm-g-SB1**

NMR spectra of SB1, SB2 and grafted polyamines

^{1}H NMR (D_2O, 300 MHz):

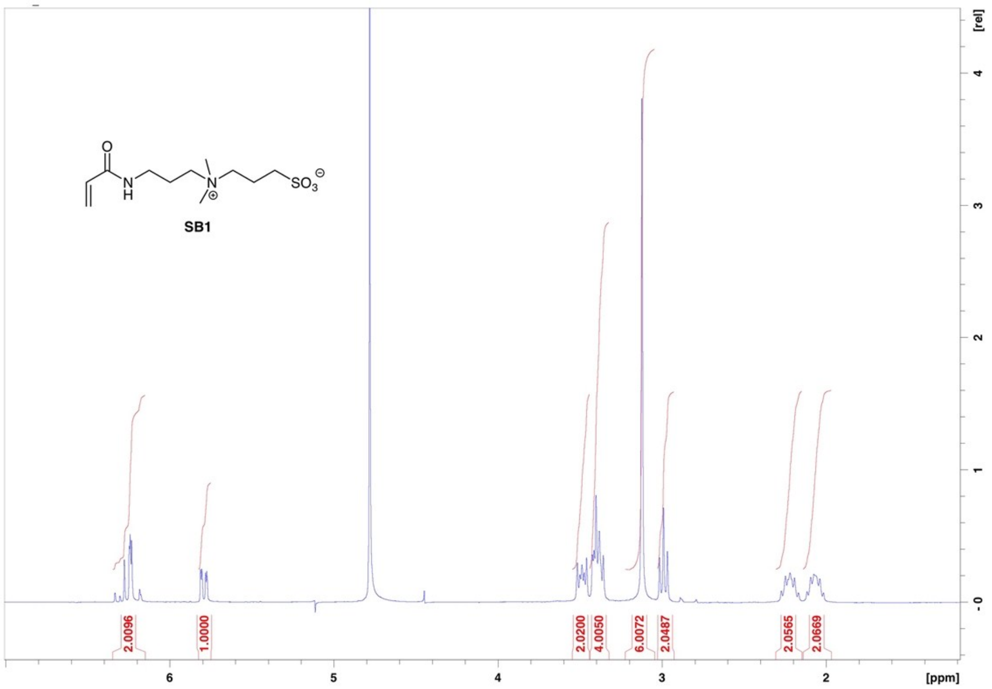

^{13}C NMR (D_2O, 75 MHz):

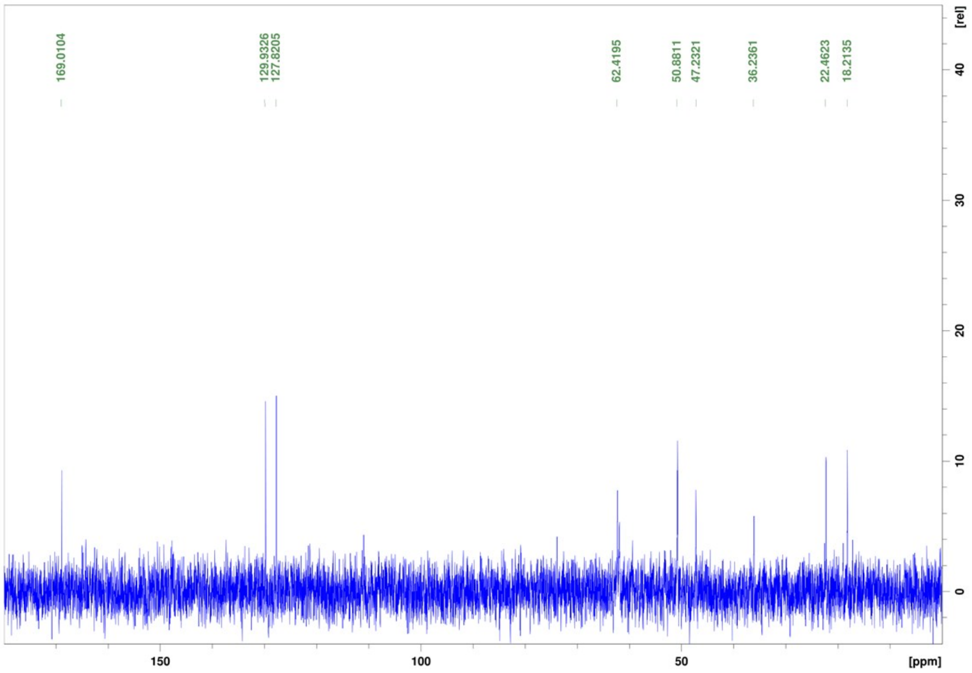

Figure S3. 1H and 13 C NMR spectra of sulfobetaine **SB1**.

^{1}H NMR (D_2O, 300 MHz):

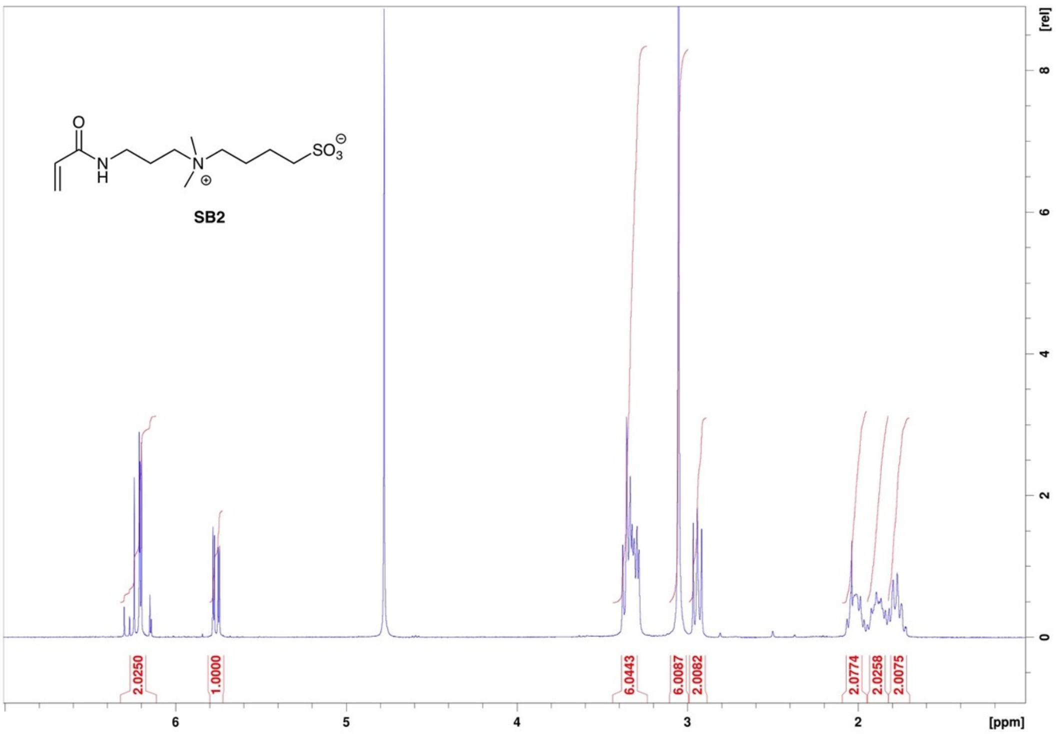

^{13}C NMR (D_2O, 75 MHz):

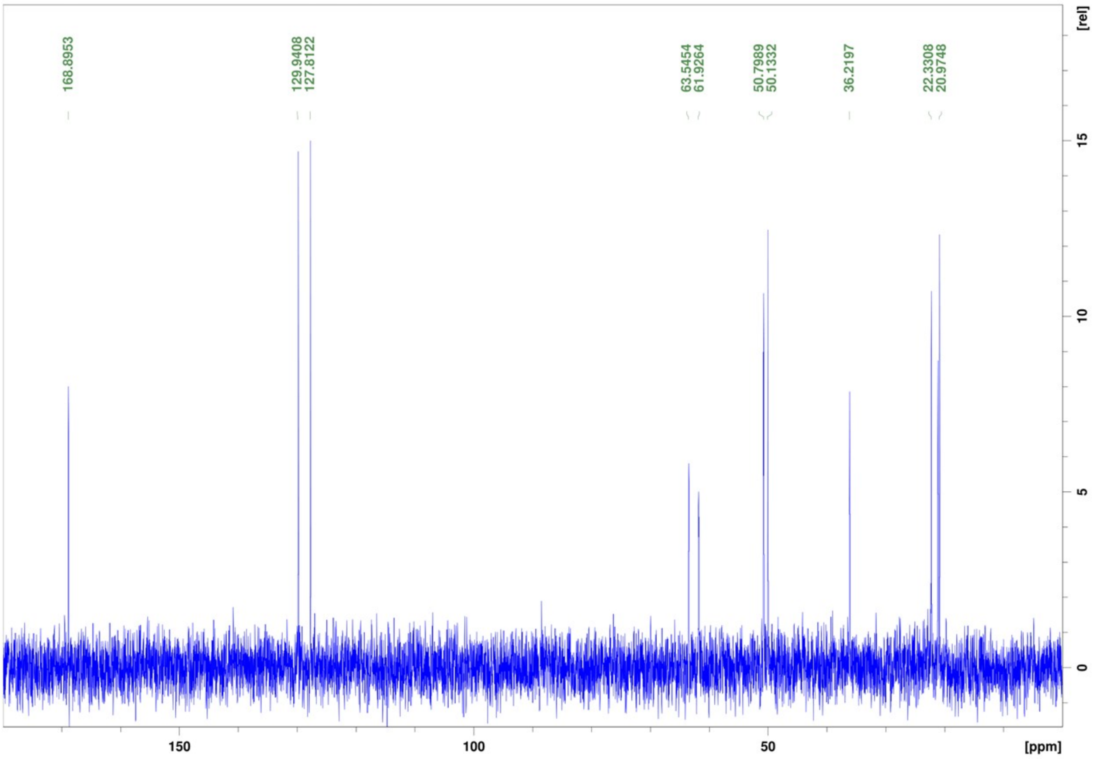

Figure S4. 1H and 13 C NMR spectra of sulfobetaine **SB2**.

PVAm-g-SB1 (DS = 22%)

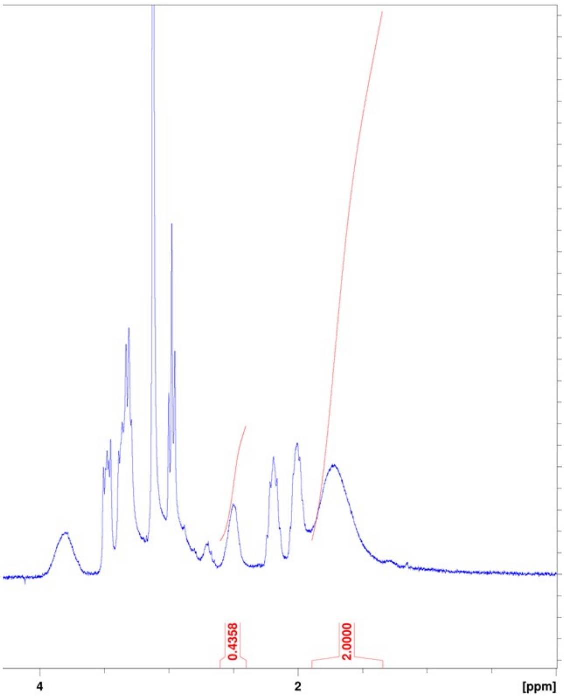

PVAm-g-SB1 (DS = 41%)

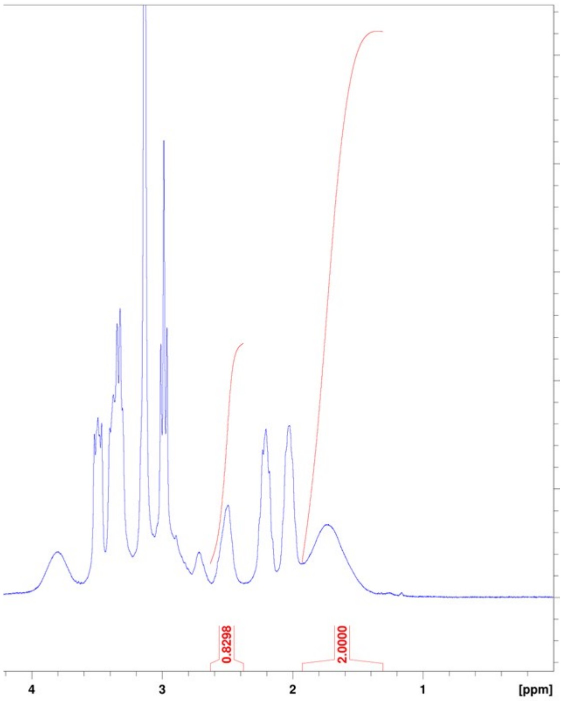

PVAm-g-SB1 (DS = 63%)

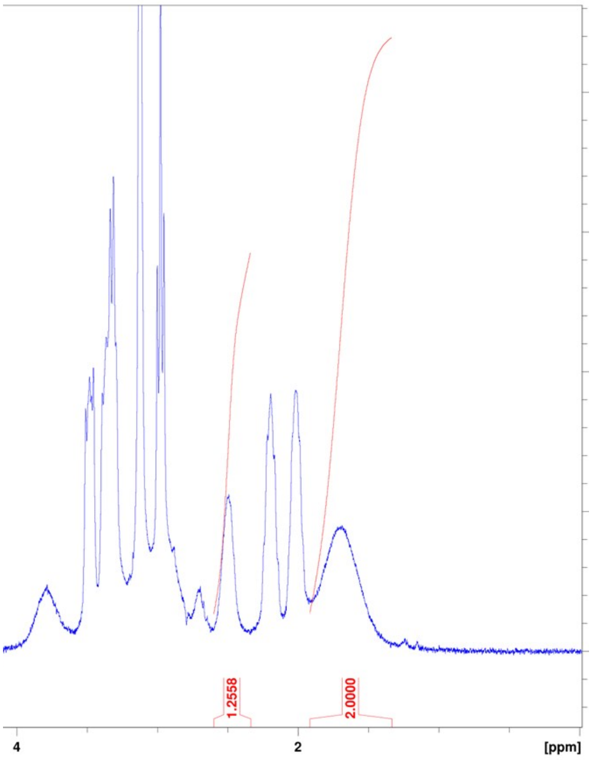

PVAm-g-SB1 (DS = 82%)

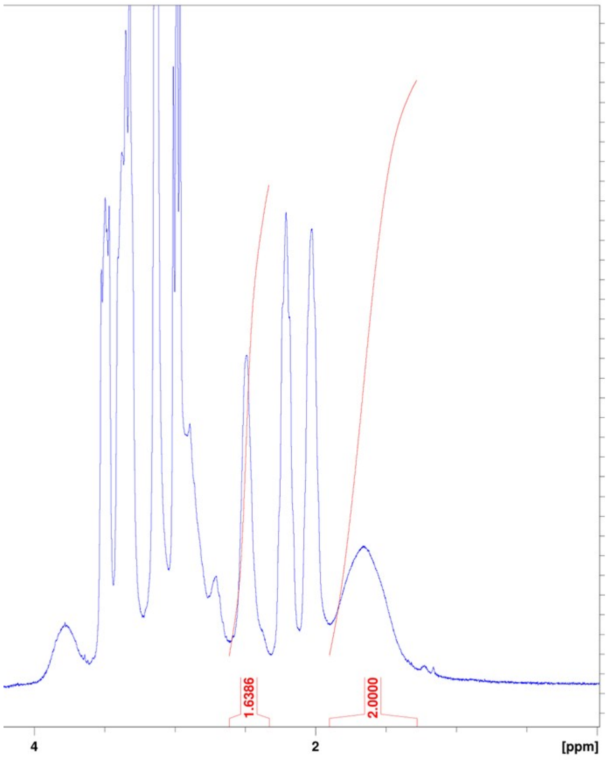

Figure S5. Relevant parts of the ^{1}H NMR spectra (D_2O, 300 MHz) of **PVAm-g-SB1** used for the determination of the DS value.

PVAm-g-SB2 (DS = 18%)

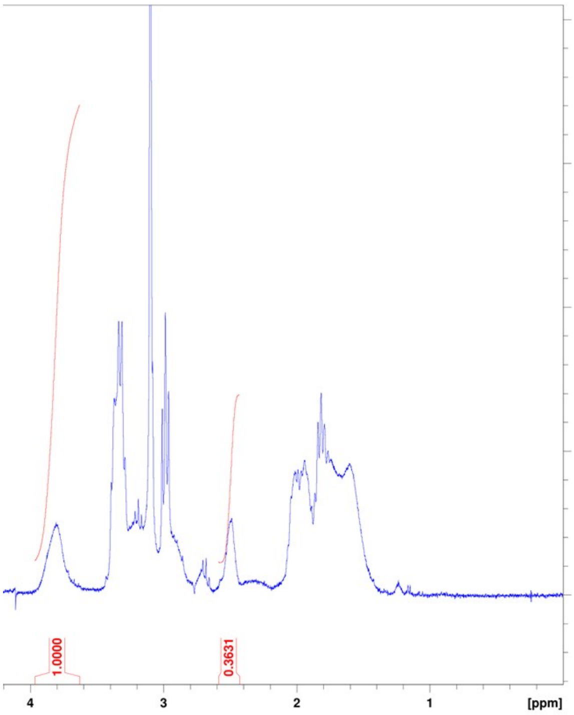

PVAm-g-SB2 (DS = 40%)

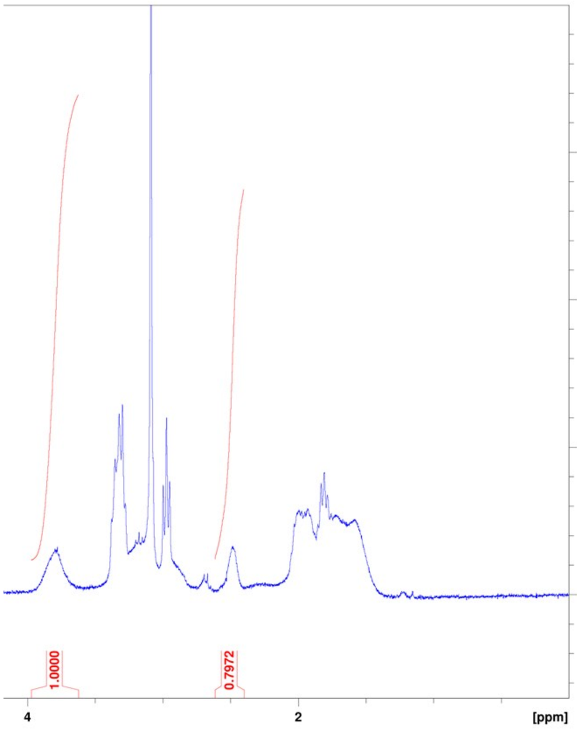

PVAm-g-SB2 (DS = 60%)

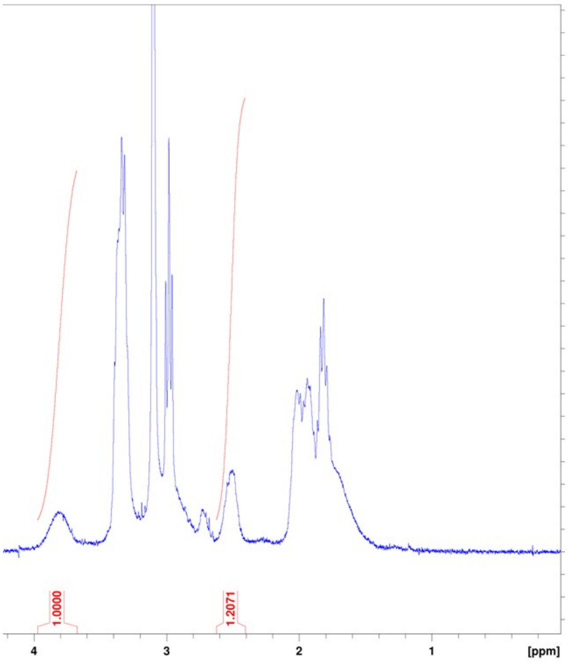

PVAm-g-SB2 (DS = 86%)

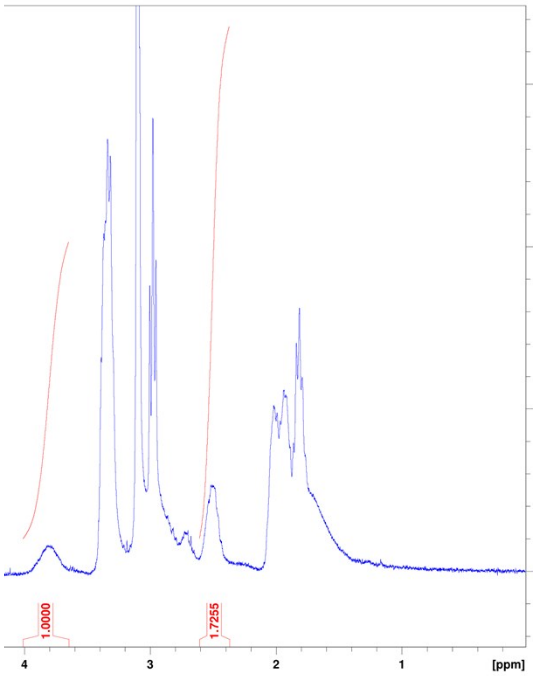

Figure S6. Relevant parts of the ^1H NMR spectra (D_2O, 300 MHz) of **PVAm-g-SB2** used for the determination of the DS value.

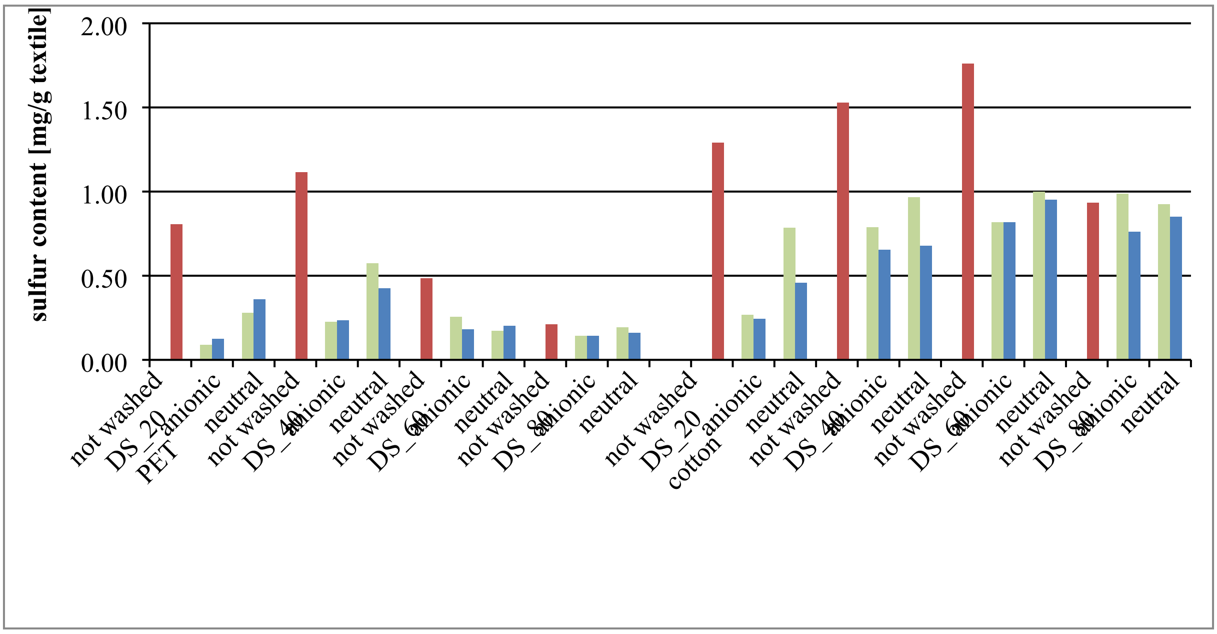

Figure S7. Sulfur content of PET and cotton finished with **PVAm-g-SB2** with different DS.

The sulfur content was determined by ICP-OES. The fabrics were finished with **PVAm-g-SB2** with different degrees of substitution (DS) and then used either directly after rinsing with water (not washed, red bars) or after 1 (green bars) and 5 (blue bars) wash cycles according to DIN EN ISO 105-C06 using an anionic and neutral detergent (washed).

With the ICP results, it is possible to determine the exact add-on of the polymer on the textile (see Figure 3). First of all, the quantity of sulfur (m) has to be calculated from the obtained sulfur mass (m):

$$n\,(sulfur) = \frac{m\,(sulfur)}{M\,(sulfur)}$$

For each DS the average molar mass (MØ) of the polymer per repeat unit was determined with the help of ChemDraw.

	SB2	SB1
DS_20	$M_Ø$ = 101.7 g / mol	$M_Ø$ = 98.7 g / mol
DS_40	$M_Ø$ = 160.2 g / mol	$M_Ø$ = 154.3 g / mol
DS_60	$M_Ø$ = 218.7 g / mol	$M_Ø$ = 209.9 g / mol
DS_80	$M_Ø$ = 277.2 g / mol	$M_Ø$ = 265.6 g / mol

With the MØ (polymer) m (polymer) can now be calculated by the following formula:

$$m\,(polymer) = \frac{n\,(sulfur) * M_{Ø}\,(polymer)}{\frac{DS}{100}}$$

DS / 100 indicates here the ratio of sulfur-containing repeat units to non-sulfur-containing repeat units.

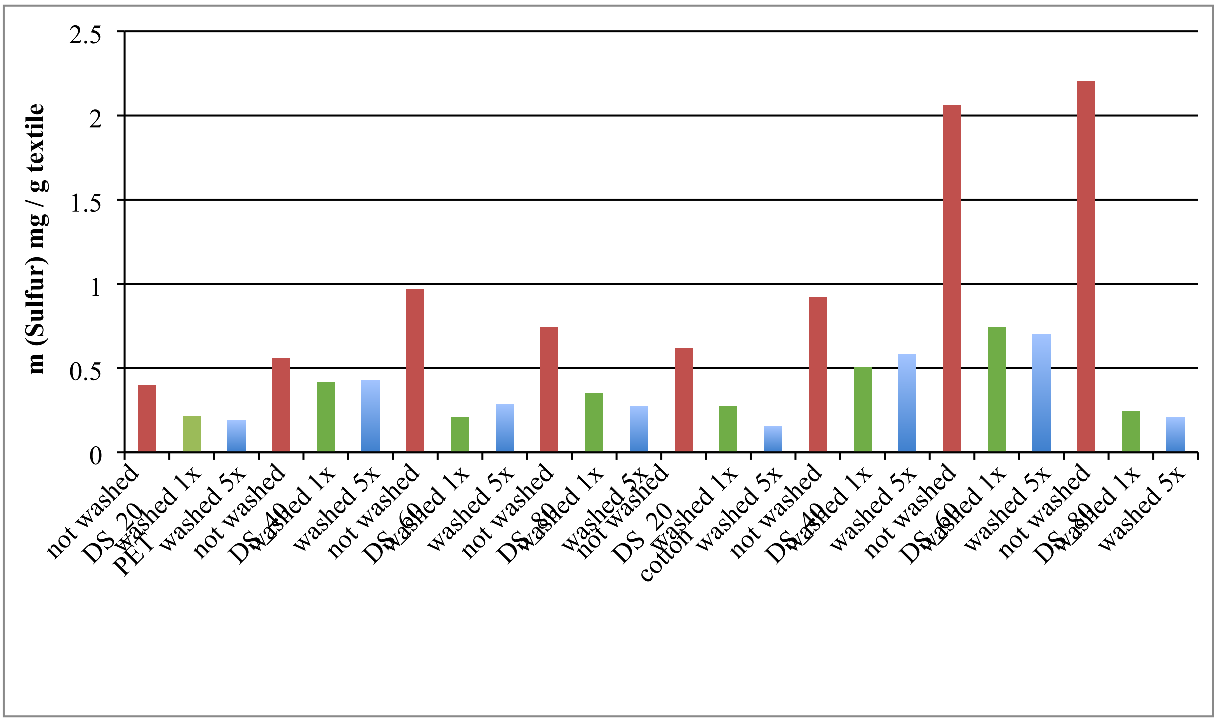

Figure S8. Sulfur content of PET and cotton finished with **PVAm-g-SB1** with different DS.

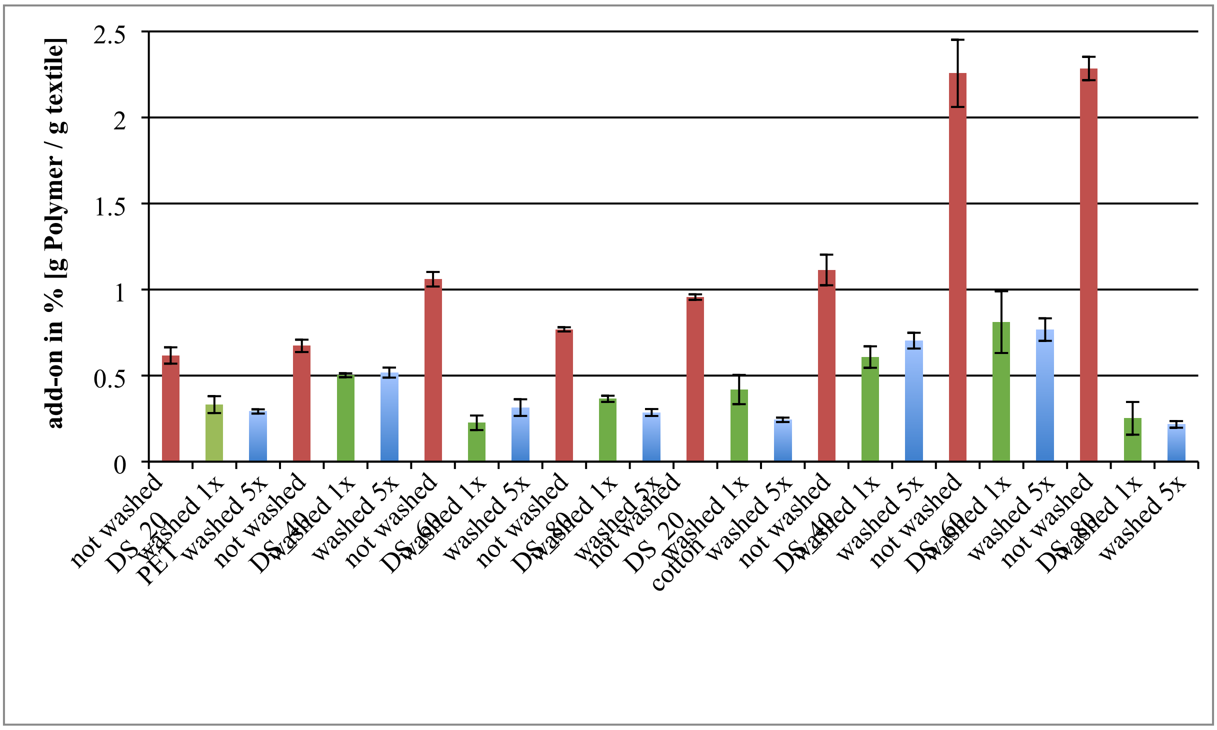

Figure S9. Add-on of PET and cotton finished with **PVAm-g-SB1** with different DS.

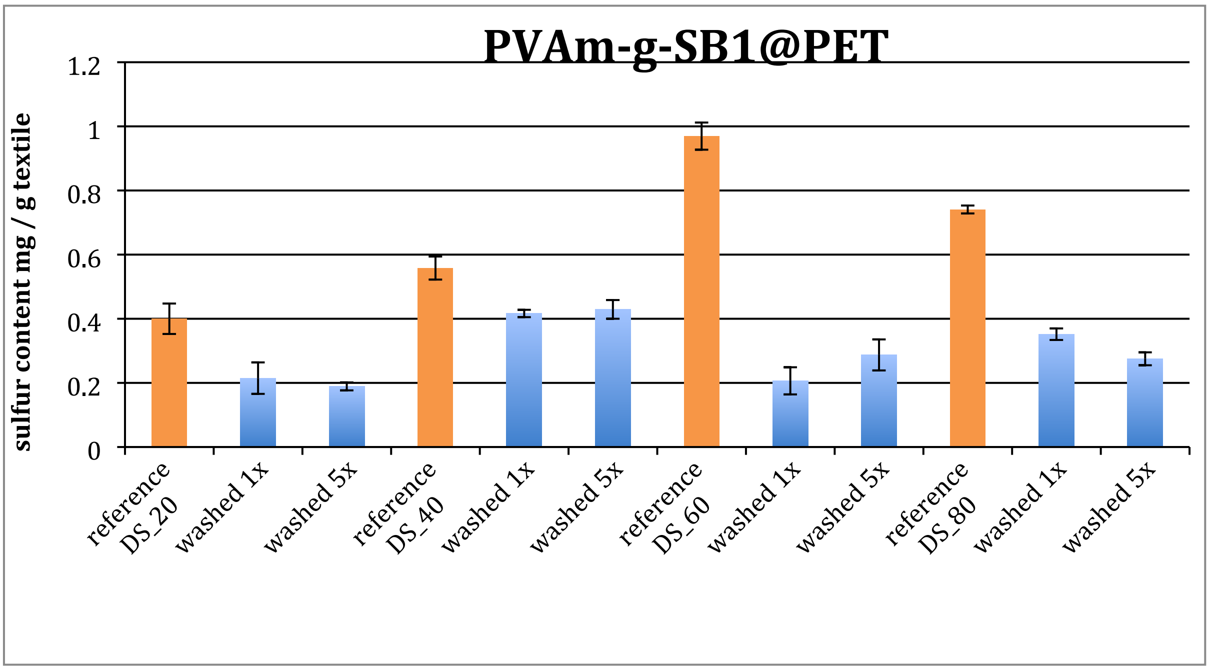

Figure S10. Sulfur content for **PVAm-g-SB1 on PET** after finishing, 1 and 5 wash cycles. The samples were washed according to DIN EN ISO 105-C06 using ECE detergent.

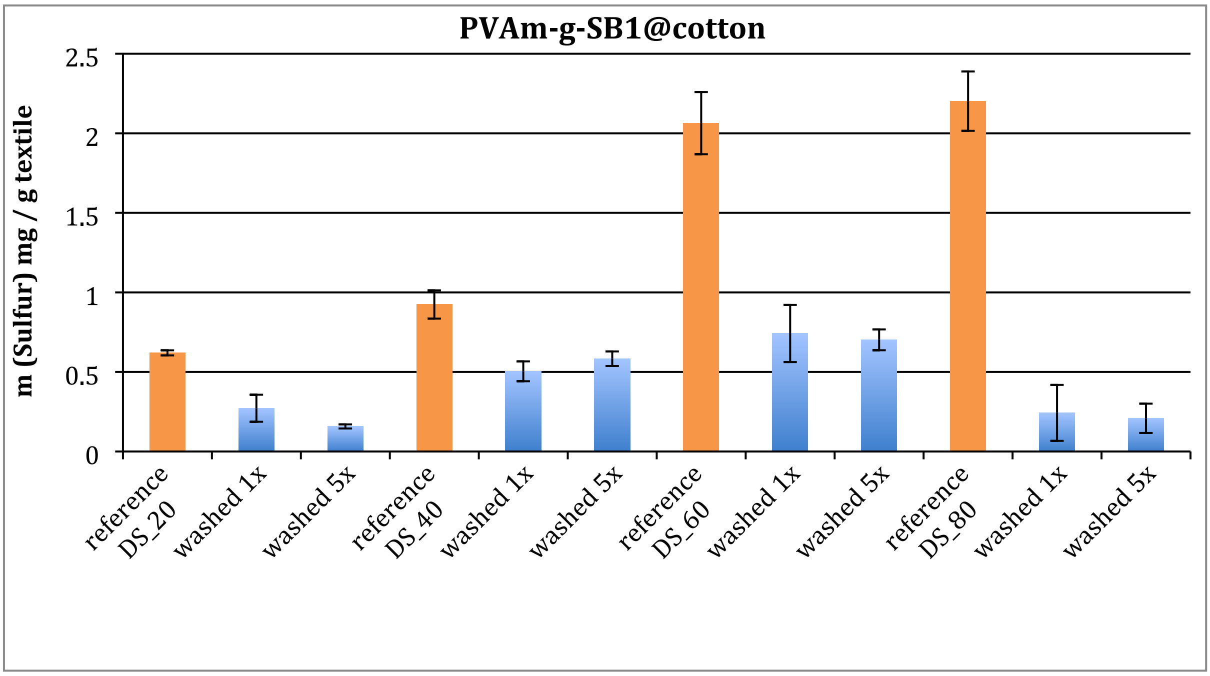

Figure S11. Sulfur content for **PVAm-g-SB1 on cotton** after finishing, 1 and 5 wash cycles. The samples were washed according to DIN EN ISO 105-C06 using ECE detergent.

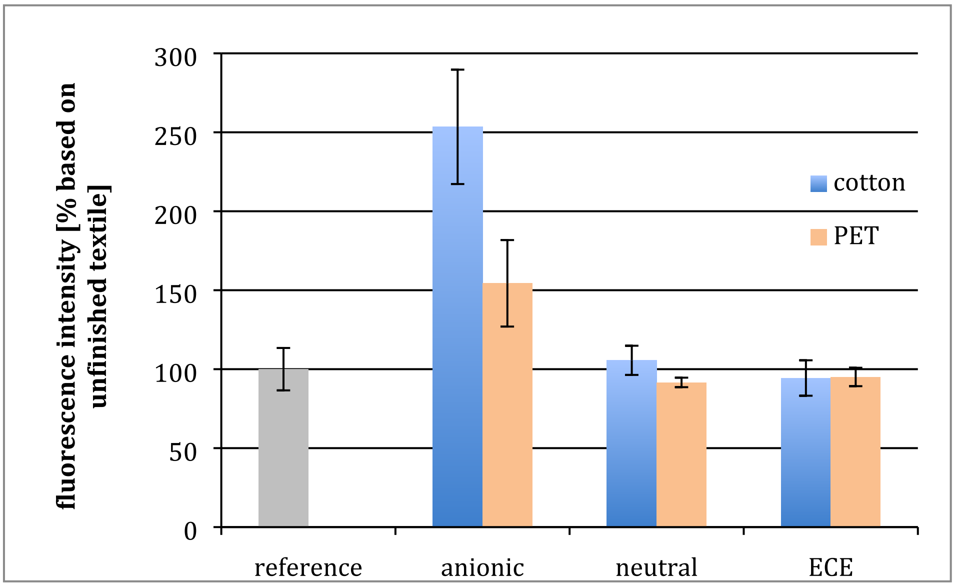

Figure S12. Results of the protein adhesion tests for **PET and cotton** washed with according to DIN EN ISO 105-C06 using different detergents.

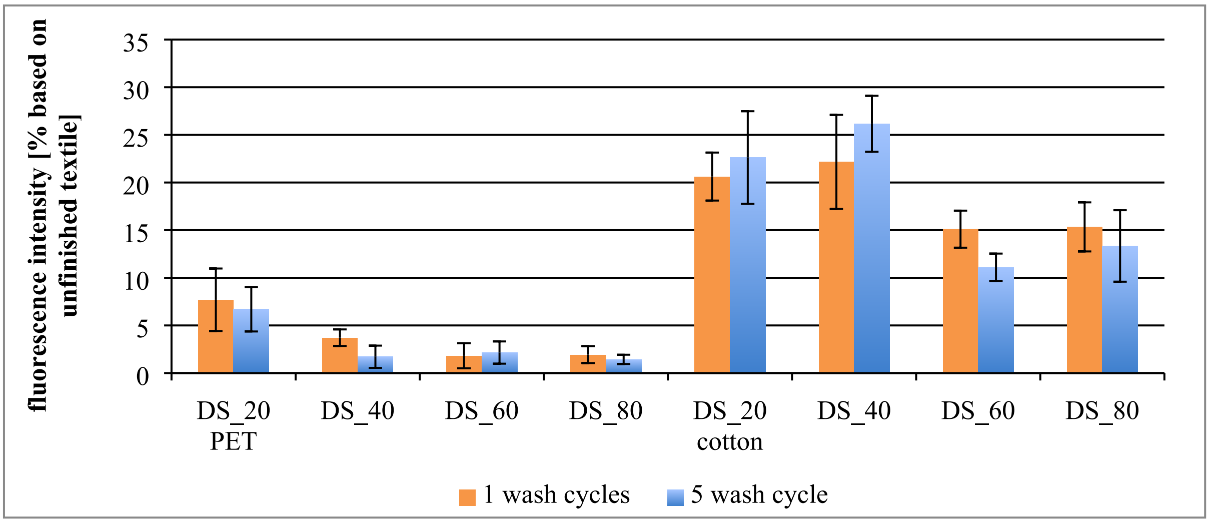

Figure S13. Results of the protein adhesion tests for **PVAm-g-SB2** after 1 and 5 wash cycles. The samples were washed according to DIN EN ISO 105-C06 using ECE detergent.

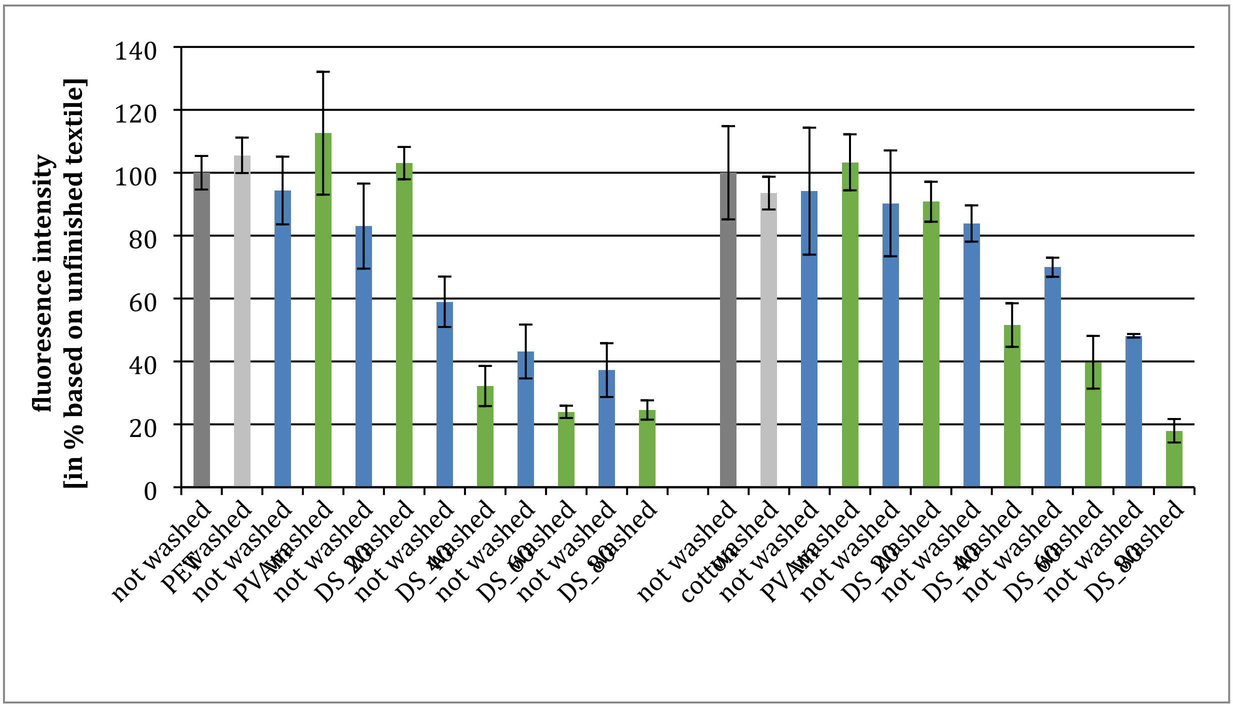

Figure S14. Results of the protein adhesion tests for **PVAm-g-SB1** after finishing and 5 wash cycles. The samples were washed according to DIN EN ISO 105-C06 using ECE detergent.

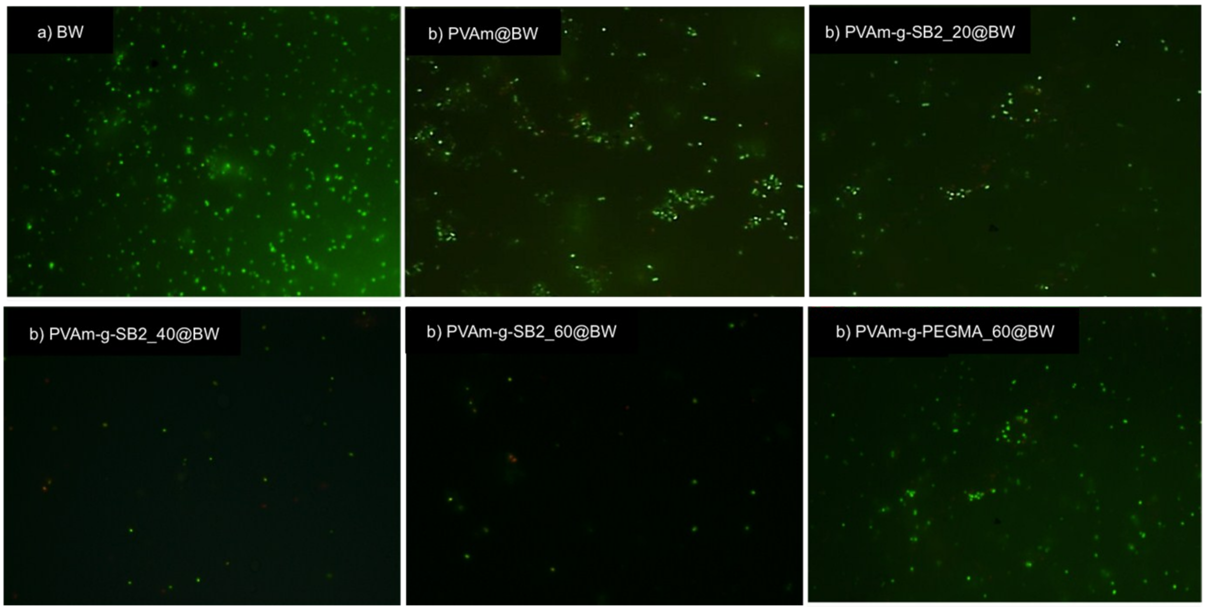

Figure S15. Fluorescence micrographs of different cotton fabrics after incubation with *E. coli* cell suspensions and staining (green: live, red: dead). a) untreated; b) finished with PVAm; c-e) finished with sulfobetaine-modified PVAm with DS 20, 40, and 60%, respectively; f) finished with PEG-modified polyvinylamine with DS=60%.

Determination of antimicrobial activity after washing

Escherichia coli pGLO: The *E. coli pGLO* was inoculated from the culture plate into a test tube with 5 mL LB medium (ampicillin and arabinose addition) and incubated for 24 h at 37°C and 180 rpm. Subsequently, the culture was diluted to such an extent that the CFU was about 10^{4-5}. Textile pieces (d = 15 mm) were placed with sterile tweezers in a 24 well plate. 100 µL of medium were added to completely moisten the textile and then 100 µL of diluted preculture were added. The well plate was closed with an air-permeable film and incubated at 37°C and 120 rpm for maximum 48 h. The fluorescence intensity was read out every hour with a plate reader. In case the test was carried out for longer than 24 h, further 100 µL of medium were added to prevent the sample from drying out

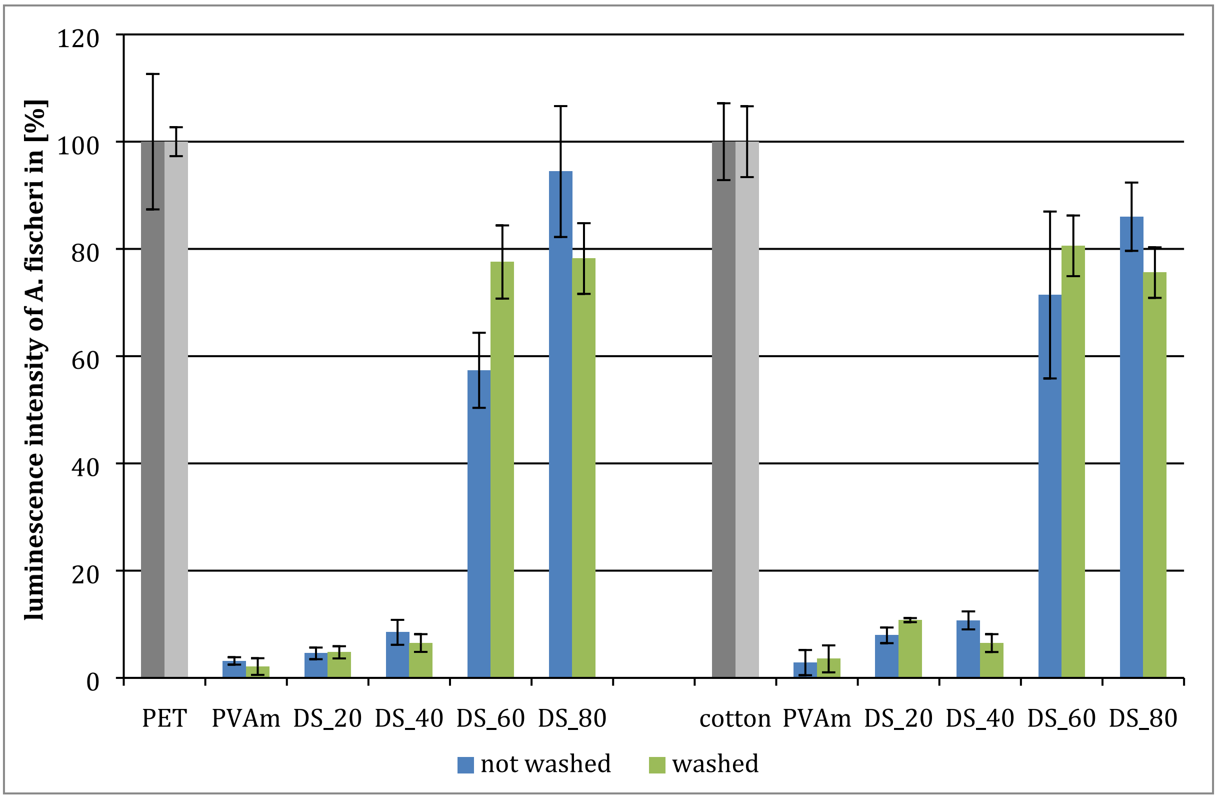

Figure S16. Antibacterial activity before and after 5 wash cycles against the gram-negative strain A. fischeri of different PET and cotton fabrics modified with **PVAm-g-SB2** with different DS.

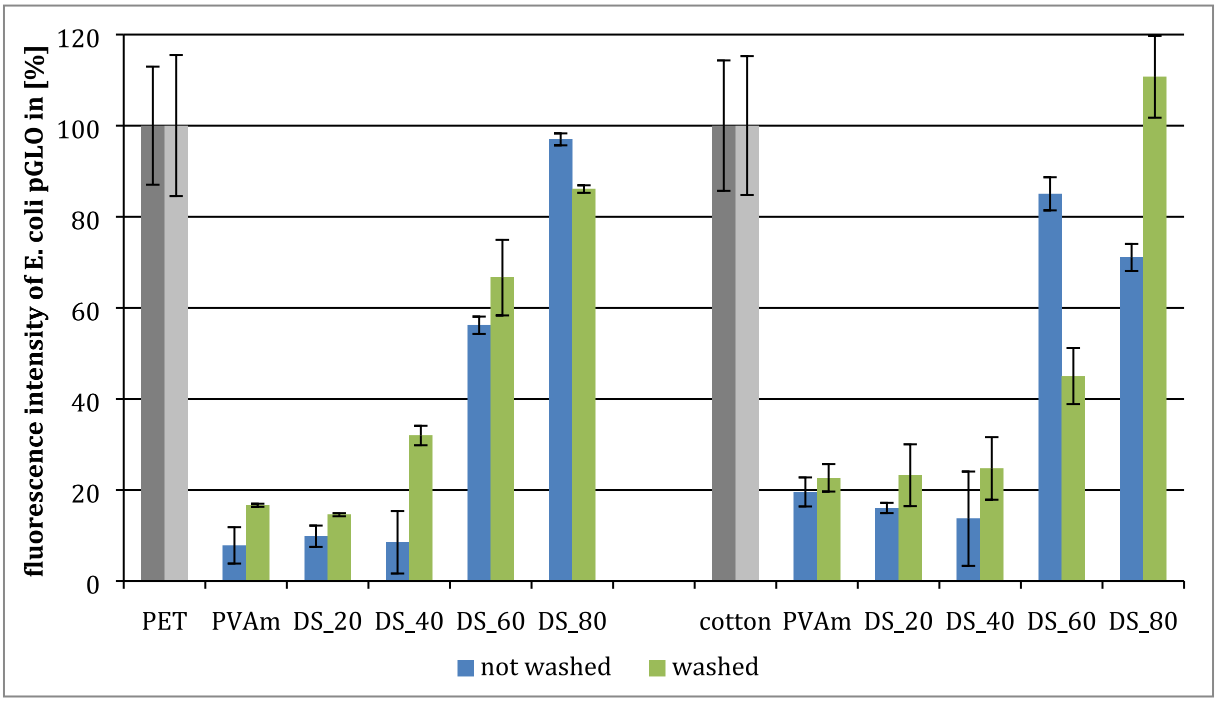

Figure S17. Antibacterial activity before and after 5 wash cycles against the gram-negative strain E. coli pGLO of different PET and cotton fabrics modified with **PVAm-g-SB2** with different DS.

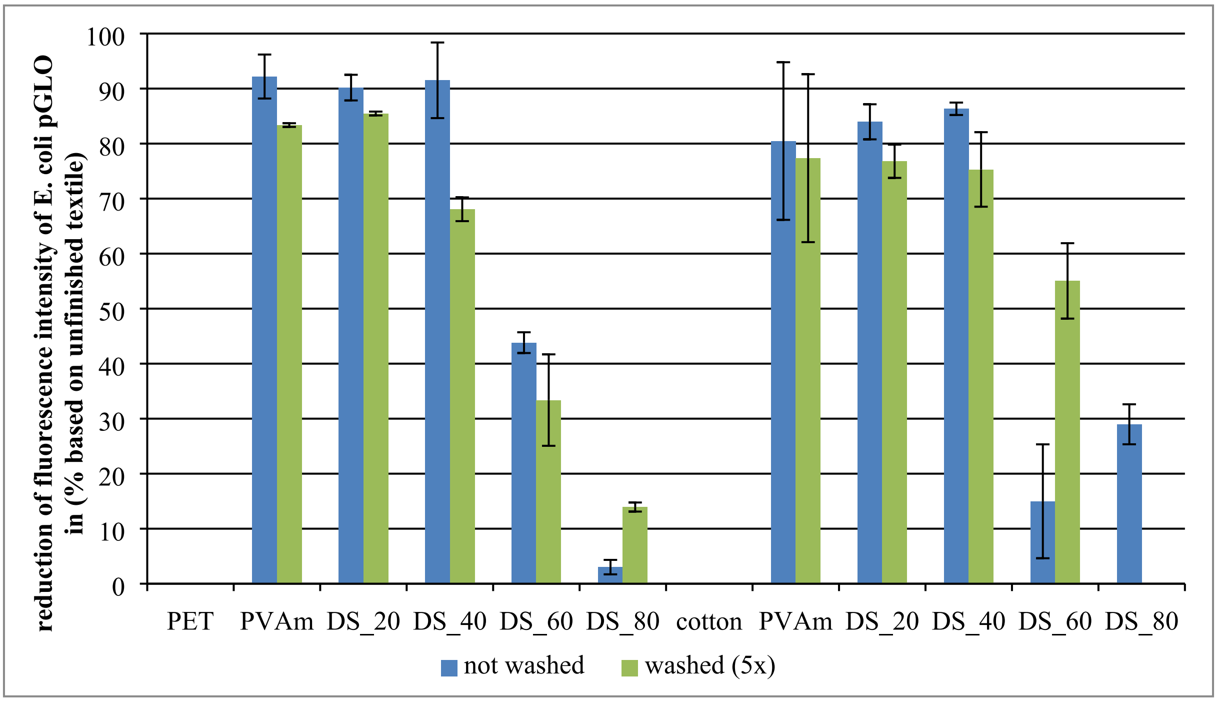

Figure S18. Reduction of fluorescence intensity before and after 5 wash cycles against the gram-negative strain E. coli pGLO of different PET and cotton fabrics modified with **PVAm-g-SB2** with different DS.
